# Reliability-Audited Explainable AI for Multi-Class Skin-Lesion 2 Triage Under Cross-Device Domain Shift

**DOI:** 10.3390/diagnostics16203368

**Published:** 2026-10-16

**Authors:** Hayder Makki Shakir, Naseer Al-Imareen, Muslem Al-Saidi, Maroua Zalfani

**Affiliations:** 1 Computer Information Systems, University of Al-Qadisiyah, Al-Diwaniyah 58001, Al-Qadisiyah, Iraq; hayder.m.shakir@qu.edu.iq; 2 Department of Computer Science, University of Al-Qadisiyah, Al-Diwaniyah 58001, Al-Qadisiyah, Iraq; naseer.jabbar@qu.edu.iq; 3 College of Computer Science and Information Technology, Wasit University, Al-Rabee District, Al Kut 52001, Wasit Governorate, Iraq; muslem@uowasit.edu.iq or muslem@sze.hu; 4 Department of Automation, Széchenyi István University, Egyetem Tér 1, 9026 Gyor, Hungary; 5 Doctoral School of Multidisciplinary Engineering Sciences, Széchenyi István University, 9026 Gyor, Hungary

**Keywords:** skin-lesion classification, cross-device domain shift, reliability auditing, uncertainty quantification, conformal prediction, out-of-distribution detection, explainable artificial intelligence

## Abstract

**Background/Objectives:** Reliable skin-lesion AI requires evaluation beyond the development distribution. This study assessed whether a source-developed multi-class system preserves discrimination and reliability when transferred to an unseen smartphone-acquired domain. **Methods:** A source-only multimodal four-class classifier was developed using HAM10000 and Derm7pt and evaluated on PAD-UFES-20 without target-domain adaptation. Reliability was assessed using class-specific calibration, uncertainty-based error detection, class-conditional conformal prediction, selective classification, demographic subgroup auditing, controlled ablation, and an independent DDI out-of-distribution (OOD) and explainability (XAI) audit. **Results:** Across five prespecified seeds, macro-F1 decreased from 74.27 ± 1.36% on the source domain to 54.92 ± 2.60% on PAD-UFES-20. Calibration, conformal efficiency, and selective coverage also deteriorated externally, although macro-AUROC remained comparatively preserved. The independent DDI audit showed limited OOD separation, while explanation stability was interpreted only as descriptive evidence. Ablation analysis indicated that clinical metadata contributed importantly to external performance. **Conclusions:** Internally reproducible discrimination did not guarantee reliable external behavior. These findings support explicit external reliability auditing and specialist-supervised selective decision support before cross-device deployment.

## 1. Introduction

Artificial intelligence (AI) has shown great promise in assisting the evaluation of skin lesions by learning discriminative representations directly from medical images. Deep neural networks have demonstrated excellent diagnostic performance in controlled dermoscopy image settings and have achieved performance comparable to that of experienced dermatologists on selected tasks [1]. However, good performance with high discrimination on a prespecified test set does not imply the generalizability of a model to a different patient population, imaging device, acquisition environment, or clinical workflow. Therefore, when considering AI-assisted skin-lesion assessment, predictive performance must be evaluated alongside generalizability, uncertainty, fairness, interpretability, and the ability to recognize cases for which an automated recommendation may be insufficiently reliable. The availability of large public datasets has accelerated research on automated multi-class skin lesion classification. HAM10000 presented a multi-source collection of 10,015 dermoscopy images of seven common diagnostic categories and became a major benchmark for model development thereafter [2]. The use of images from multiple sources increased the dataset’s diversity, but the common reliance on fixed benchmark partitions also encouraged the optimization of performance within a limited dermoscopy image domain. At the same time, multimodal learning has demonstrated that fusing image representations with structured clinical information can improve lesion characterization by adding diagnostic context that may not be captured entirely by visual appearance alone [3]. These developments laid important foundations for multi-class dermatological AI but did not eliminate the dependence on dataset-specific acquisition patterns or population characteristics.

The envisioned clinical role of AI has also evolved from isolated image classification to collaborative decision support. Evidence suggests that classification performance may benefit from combining human assessment with algorithmic output rather than relying solely on one component [4]. Moreover, it has been demonstrated that deep learning systems can support differential diagnosis across skin conditions and are not limited to binary melanoma recognition [5]. Studies of human–AI collaboration have demonstrated that algorithmic support, when properly designed, can enhance diagnostic decisions and may be especially valuable for less experienced clinicians [6]. These results both support the use of AI as an assistive mechanism for triage and emphasize the necessity of presenting predictions in a way that communicates reliability rather than encouraging blind acceptance of model output. The discrepancy between controlled dermoscopy image data and the heterogeneity of images encountered in routine clinical practice is a major challenge for reliable application. PAD-UFES-20 offers clinical skin lesion images obtained with different smartphone devices, together with patient-related information, which entails variations in illumination, image resolution, background content, viewing distance, and acquisition hardware [7]. These differences represent a clinically relevant cross-device domain shift that could reduce the validity of representations learned solely from dermoscopy images. Acquisition robustness can be considered at two complementary levels: reducing image-quality variability before classification and auditing whether predictive reliability is preserved after transfer to a new acquisition setting. Also, patient-centered datasets with images and metadata have highlighted the need to preserve the patient-level structure during model development and evaluation [8]. Therefore, random image-level partitions within a single dataset may overestimate generalization when images associated with related lesions or patients are not well separated across experimental subsets. Rapid methodological progress notwithstanding, several barriers continue to hinder the translation of dermatological AI into heterogeneous clinical environments. These include poor dataset representativeness, inconsistent acquisition protocols, class imbalance, insufficient external validation, and uncertainty about model behavior outside the development distribution [9]. However, extensive benchmarking has shown that strong internal performance does not necessarily imply similar reliability under variations in data composition, disease prevalence, or imaging conditions [10]. Therefore, evaluation using only accuracy or the area under the receiver operating characteristic curve may provide an incomplete picture of the model’s reliability, especially when the target setting differs from the data used for optimization. Prospective evaluation is beginning to provide more realistic evidence of how AI-assisted skin-lesion systems perform. The PROVE-AI study evaluated open-source dermoscopy-based AI under prospective conditions and highlighted the importance of validating models beyond retrospective benchmark partitions [11]. Studies on a range of skin tones have also suggested that the effect of AI support may differ by demographic group and clinical setting [12]. Multicenter evaluation has reinforced the need to evaluate performance across institutions, acquisition settings, and patient cohorts rather than assuming that internal discrimination will be maintained after deployment [13]. Human–AI interaction studies show that the clinical effect of algorithmic assistance depends not only on model performance but also on how users communicate and interpret predictions [14]. Taken together, these results suggest that external performance, subgroup reliability, and interaction-aware use should be considered as primary evaluation objectives rather than secondary analyses. The increasing size and diversity of dermatological image repositories offer new opportunities for cross-domain reliability assessment. Datasets like BCN20000 contain lesions acquired under less standardized real-world conditions, thereby exposing models to variations not well represented in highly curated collections [15]. More recent multi-source resources, such as DERM12345, increase the number of diagnostic subclasses and the heterogeneity of the appearance and acquisition origin of the lesions [16]. However, increasing the number of images or classes does not necessarily ensure equitable model behavior. Public skin lesion datasets often lack complete skin type information and have small sample sizes for some demographic groups [17]. Thus, high performance at the population level may be consistent with clinically important differences in sensitivity, false-negative rates, or confidence reliability across subgroups. Domain-generalization methods are therefore needed to learn representations that remain informative as acquisition conditions change. A systematic review of medical-image classification has demonstrated that domain shift is a consistent cause of performance degradation and highlighted the need for evaluation protocols that clearly separate source-domain optimization from testing on unseen domains [18]. This is particularly important in skin-lesion analysis, where image appearance may vary due to device characteristics, illumination, magnification, clinical setting, and population composition. Hence, a thorough assessment should determine whether a classifier preserves discrimination on an unseen domain, as well as whether the transfer affects the classifier’s predictive confidence and subgroup behavior.

Estimates of uncertainty and explainability provide complementary information to assess this reliability. Conventional neural classifiers can assign high-confidence probabilities to ambiguous, out-of-distribution, or distribution-shifted inputs. Hence, probability calibration is needed to ensure that the confidence in the prediction matches the correctness observed. Uncertainty-aware inference can also detect cases in which an unrestricted automated recommendation should not be provided. Explainable artificial intelligence can highlight image areas associated with a prediction, but visually convincing attribution maps do not necessarily indicate that the model is built on stable or clinically meaningful evidence. Therefore, explainability and uncertainty should be assessed together rather than as standalone indicators of trustworthiness [19]. Recent evidence syntheses have also stressed that good aggregate performance is not enough to demonstrate clinical reliability of AI-based skin-cancer assessment [20]. However, prior work typically treats multi-class classification, domain generalization, demographic fairness, probability calibration, uncertainty estimation, and explainability as separate goals. Models trained on a single dermoscopy image benchmark can achieve good internal discrimination without investigating whether calibrated confidence, subgroup performance, and fidelity of explanation are maintained after transfer to heterogeneous clinical or smartphone-acquired images. Existing explainability approaches often provide attribution maps without assessing their robustness to acquisition-induced changes, and uncertainty estimates are seldom integrated with subgroup-specific calibration and explanation robustness in a holistic triage framework. Moreover, many systems produce a prediction for every input without an explicit mechanism to defer unfamiliar, poorly calibrated, or explanation-unstable cases to specialist assessment.

This study addresses cross-device reliability as an evaluation problem rather than assuming that strong internal discrimination determines deployment readiness. The primary source-only multi-class pipeline evaluates discrimination, probability calibration, uncertainty-based error detection, class-conditional conformal prediction, demographic subgroup reliability, and selective classification. An independent post-development DDI audit separately evaluates OOD separation and explanation stability using a fixed, prespecified checkpoint of the primary diagnostic architecture. The DDI OOD and XAI outputs are reported only as complementary audit evidence and are not used for probability calibration, conformal fitting, selective-threshold optimization, or accept/review/defer decisions. Demographic analyses are restricted to those sufficiently represented in the available metadata and are interpreted as subgroup reliability audits rather than as evidence that demographic fairness or equity has been achieved. The implemented selective-classification pathway therefore relies only on source-fitted predictive-reliability quantities derived from the frozen primary model, whereas OOD and explanation-stability analyses remain methodologically independent post-development audits. The main contributions are as follows:

1. We establish a strict source-only cross-device evaluation protocol, with HAM10000 and Derm7pt being used for development and PAD-UFES-20 as a completely unseen smartphone-acquired evaluation domain.
2. We evaluate predictive reliability beyond accuracy using class-specific probability calibration, uncertainty-based error detection, class-conditional conformal prediction, and selective classification.
3. Demographic reliability is audited through subgroup-specific discrimination and calibration measures for supported attributes based on the available metadata, without claiming that demographic fairness has been achieved.
4. As a secondary post-development audit, we evaluate unsupported-diagnosis OOD separation and perturbation stability of Grad-CAM++ and Integrated Gradients using independent DDI data.

**Figure 1:**
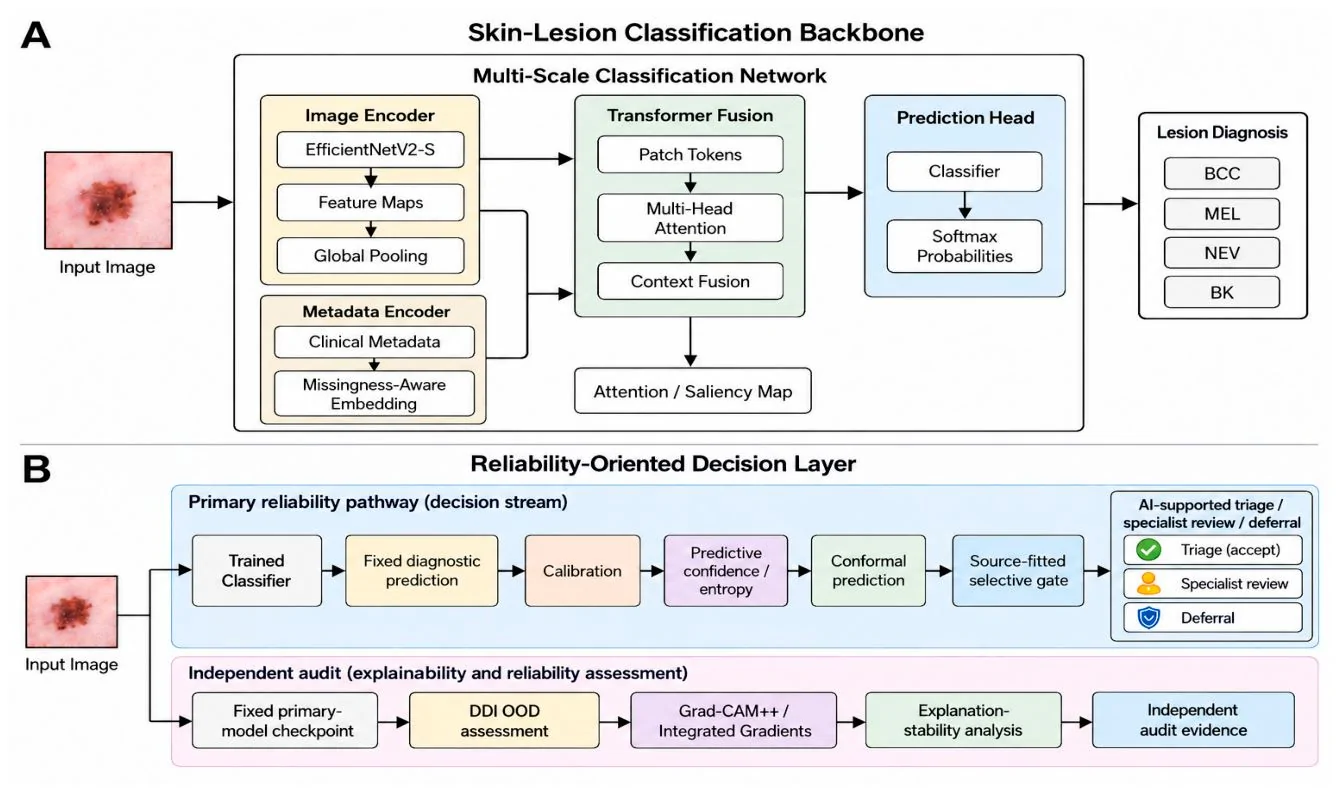
Conceptual overview of the primary source-fitted reliability pathway and independent post-development reliability audit. The primary selective-classification pathway uses source-fitted calibration, predictive confidence/entropy, and class-conditional conformal information. DDI-based OOD assessment and explanation-stability analysis are performed independently using a fixed checkpoint of the primary diagnostic architecture and do not modify the selective-classification decisions.

In this work, we incorporate these components into a common evaluation framework to push the goal of multi-class skin-lesion AI from maximizing internal classification performance to achieving reliable and interpretable behavior while explicitly auditing subgroup-specific performance under external dataset shift. The overall organization of the proposed framework is summarized in Figure 1. As shown in Figure 1A, the primary diagnostic backbone integrates visual and clinical-metadata information to generate four-class predictions. Figure 1B presents the reliability-oriented decision layer, separating the source-fitted selective-classification pathway from the independent post-development OOD and explanation-stability audit.

## 2. Materials

The early deep learning studies on skin-lesion classification primarily focused on improving the discriminative power of features on curated dermoscopy image benchmarks. By combining residual feature extraction with attention mechanisms, attention residual learning improved the representation of diagnostically informative regions [21]. Patch-based attention and diagnosis-guided loss weighting then improved local representation and reduced the effect of class imbalance [22]. Such approaches made progress on benchmark-level discrimination, but the reliability of their evidence still relied on the quality, diversity, and documentation of the datasets used for their development and evaluation. Major deficiencies were noted in dataset transparency, verification of diagnostic labels, and reporting of patient diversity following a systematic audit of dermatology AI datasets [23]. The lack of information on ethnicity, race, and skin tone hampered the ability to assess whether the reported model performance was transferable across patient populations. These findings indicate that architectural enhancements cannot compensate for poorly characterized development data, and that overall benchmark performance should not be interpreted as evidence of fair generalization. Then, joint segmentation–classification learning leveraged the complementary relationship between lesion localization and diagnostic prediction [24]. Imbalance-aware single-model learning enhanced performance with limited and unequally distributed training data [25]. These methods addressed important representation and optimization issues but were mainly evaluated on internal or closely related dermoscopy image datasets. Therefore, they did not assess whether improved feature learning could yield robust performance across different clinical image domains. The Diverse Dermatology Images evaluation directly demonstrated that benchmark performance may not generalize across populations and disease distributions [26]. State-of-the-art dermatological AI models performed poorly on darker skin types and rare diseases, demonstrating that aggregate performance can mask clinically relevant subgroup differences. It also found that adapting to diverse images can reduce performance differences, underscoring the need for representative data. However, adaptation to seen target examples differs from generalization to an unseen acquisition domain. Therefore, the remaining problem is not only to increase dataset diversity but also to determine whether the models’ discrimination and confidence remain reliable before the target-domain data are available.

To enhance the features and predictive robustness, ensemble and multimodal methods were introduced. Transfer learning across multiple scales and networks fused complementary representations from different architectures and input resolutions [27]. Multi-resolution EfficientNets ensemble further integrated patient metadata and an additional unknown-category mechanism [28]. These methods improved the use of visual and contextual information but did not establish whether ensemble confidence remained calibrated after transfer to an unseen acquisition environment. Model averaging can reduce prediction variance while still relying on source domain characteristics. SkiNet extended the scope of skin lesion analysis by integrating lesion segmentation, multi-class classification, uncertainty estimation, and post hoc explainability into a single pipeline [29]. The framework incorporated uncertainty-aware components to quantify prediction uncertainty and to produce saliency-based explanations for the model’s decisions. Its contribution shows that integrating uncertainty estimation and explainability is not per se a novel research direction. But its evaluation did not jointly address cross-device domain generalization, demographic fairness, class-wise probability calibration, conformal prediction, or explanation stability under acquisition-related changes. Therefore, the open question is whether the uncertainty and reliability of explanations still hold when explanations are transferred outside the development distribution. Transformer-based classification further enhanced the representation of global image relationships and adopted contrastive learning to improve discriminative features [30]. However, more architectural capacity does not guarantee acquisition-invariant representations or reliable probabilities. A model can achieve high internal classification performance but still be overconfident on out-of-distribution images or exploit domain-specific visual cues. Reliability under distribution shift should therefore be evaluated separately from architectural performance. Explainable AI has been extensively applied to dermatological classification to identify the image regions that contribute to model predictions. However, a systematic review showed that rigorous evaluation of explanation validity remained limited, and the clinical meaning of generated explanations was often insufficiently established [31].

Visual plausibility cannot be used to determine whether an attribution is lesion-relevant, stable in the face of non-pathological image changes, or preserved across acquisition domains. To address the issue of distribution shift, unsupervised domain adaptation methods have been used to exploit unlabeled samples from the target distribution and adapt the model [32]. Such approaches may improve target-domain performance but require that target data be available before deployment. The experimental setting differs from domain generalization, where the target acquisition domain is not available during model development and model selection. Moreover, few-shot prototypical learning has been evaluated across multiple dermoscopy image domains to reduce the requirements for target-domain supervision [33]. These studies show the value of distribution-aware representation learning but do not establish reliability in a completely unseen cross-device transfer. Their evaluation protocols did not jointly consider probability calibration, demographic disparities, and explanation stability either. Recent work has extended XAI evaluation beyond qualitative visualization. TIxAI used the difference in attribution relevance between lesion regions and surrounding image regions to measure explanation trustworthiness [34]. This offers a more objective assessment of lesion-based reasoning. However, lesion relevance and attribution stability are different properties. An explanation may remain focused within the lesion, yet vary greatly with changes in illumination, image resolution, background composition, or acquisition device. Therefore, lesion relevance cannot guarantee the reliability of explanations under cross-device distribution shift. Uncertainty quantification has been independently assessed as a tool for detecting potentially unreliable skin-lesion predictions [35]. Different uncertainty estimators can reveal predictive ambiguity that is not captured by the maximum class probability. But the estimation of uncertainty should not be confused with the calibration of probability. Calibration refers to the correspondence between the probability of correctness and the probability of being correct, whereas uncertainty estimation measures the model’s lack of knowledge or ambiguity. Conformal prediction answers a third reliability question by outputting prediction sets with a desired coverage level under some assumptions. These components provide complementary evidence and should be evaluated separately. AI-generated recommendations have been more widely accepted and trusted by physicians when presented with clinically structured explanations through dermatologist-like explanation systems [36]. Such findings show the value of clinically interpretable output but do not answer whether explanation quality is preserved in multi-class classification or when transferred between imaging devices. The relevant reliability question is not whether an explanation can be shown, but whether there remains consistent lesion-related evidence outside the domain of development. Recent hybrid frameworks have integrated convolutional representation learning, transformer-based global modeling, attention mechanisms, clinical metadata, and post hoc explainability. DermaScanAI [37] employed the HAM10000 dataset for seven-class classification, incorporating a dual attention mechanism and metadata fusion. The framework demonstrated the utility of combining local lesion features, global image information, and structured patient data. However, strong performance on a principal dermoscopy images benchmark did not predict whether discrimination, confidence calibration, subgroup behavior, or explanation reliability would be preserved in smartphone-acquired clinical images. Moreover, multimodal risk-prediction systems incorporate specific imaging and clinical information to improve lesion assessment [38]. These methods demonstrate that clinical variables can be incorporated into visual representations, but multimodal fusion does not automatically eliminate acquisition dependence. Specifically, there was no evaluation of the transferability of models developed on specialized 3D imaging to routine smartphone images. Thus, evidence of improved multimodal discrimination should be distinguished from evidence of cross-device robustness. Fairness-aware skin-cancer modeling has explored demographic disparities through lesion-guided attention and multi-objective optimization [39]. This work showed that predictive performance and demographic fairness may need to be optimized jointly and that aggregate metrics can hide subgroup differences. However, evidence of fairness established for a specific demographic attribute and classification task cannot automatically be generalized to all patient groups or lesion types. Fairness assessment should be limited to the attributes that are represented in the evaluated datasets, and should consider both discrimination and calibration, as similar classification accuracy does not imply similar confidence reliability. Recently, conformal uncertainty quantification has been applied to assess predictive fairness across patient demographics [40]. Prediction sets provided information on uncertainty at the individual and subgroup levels and helped identify ambiguous cases that may require specialist assessment. However, it did not develop a unified cross-device framework that jointly evaluated unseen-domain generalization, calibrated class probabilities, attribution stability, and reliability-based deferral. Additionally, conformal coverage should not be seen as a substitute for probability calibration, as the two methods measure different properties of predictive trustworthiness. Furthermore, feature fusion and explainable deep learning methods [41] have improved multi-class representation and model transparency.

The evaluation was conducted on external datasets, but the experimental design did not strictly follow a leave-one-domain-out generalization scheme, in which the target acquisition domain was not seen during model development and selection. Graph learning in a semi-supervised setting has also integrated multimodal information, entropy-based confidence, and selective pseudo-labeling [42]. Here, confidence estimation dominated pseudo-label quality, rather than enabling calibrated multi-class triage after cross-device transfer. Dataset dependence remains an important limitation, even when multiple predictors were combined. A study on dermoscopy images and clinical image datasets showed that the model’s behavior could be highly dependent on the development dataset and that ensemble learning could reduce, but not eliminate, such dependence [43]. This evidence supports evaluation protocols based on explicit source-target domain separation rather than inferring generalization from model averaging or internal cross-validation. A systematic review and meta-analysis comparing artificial intelligence algorithms with clinicians showed that AI performance was generally comparable to that of expert dermatologists in the included experimental studies [44].

However, the evidence base was characterized by significant methodological heterogeneity, and only a small number of the studies reviewed were conducted prospectively or in real-world clinical settings. These findings support the use of AI as assistive technology rather than as a fully autonomous diagnostic system. They also argue that high retrospective performance should not be considered sufficient evidence of clinical readiness. More recent evidence from clinical dermoscopy images and smartphone imaging settings has found ongoing limitations in equity and generalizability [45]. Diagnostic performance was lower in smartphone-based and non-specialist settings than in specialist settings, and lower for darker than for lighter skin tones. These results provide direct evidence that the reliability of dermatological AI is affected by the imaging modality, deployment context, and population characteristics. They also emphasize the value of external, cross-device evaluation and subgroup-stratified reporting rather than relying solely on aggregate benchmark metrics. A complementary approach to acquisition variability is to improve image quality before classification. Huang et al. [46] proposed SAVE, a spectrum-aided visual-enhancement method for AI-based skin-cancer detection, with the aim of reducing image-quality variations that may adversely affect downstream classification. This input-level strategy is complementary to the present reliability-auditing framework. Whereas SAVE seeks to reduce acquisition-related variability before prediction, the present study evaluates whether discriminations, calibration, uncertainty, conformal behavior, and selective reliability remain valid after a source-developed model is transferred to an unseen smartphone-acquired domain. The related work has made significant progress in multi-class representation learning, class imbalance handling, multimodal fusion, domain-aware transfer, explainability, uncertainty estimation, and fairness analysis. Yet these advances are still methodologically fragmented. Classifiers focused on benchmarks tend to prioritize internal discrimination; domain-adaptation techniques often need access to the target distribution; models aware of uncertainty do not always assess probability calibration; XAI approaches mainly assess visual plausibility or lesion relevance without quantifying the stability of the attribution; and fairness analyses rarely combine with cross-device generalization.

The evidence reviewed does not resolve four methodological requirements. First, it is not confirmed whether a multi-class model trained on heterogeneous dermoscopy images and clinical image sources can maintain discrimination in a completely unseen smartphone-acquired domain. Second, there is still little evidence for calibrating predictive confidence across lesion classes and available demographic subgroups after domain transfer. Third, the relevance of the lesion and its attribution stability are not jointly evaluated under acquisition-related perturbations in existing studies. Fourth, these reliability properties are not aggregated into a unified selective triage policy that sufficiently separates well-supported predictions from uncertain, distribution-shifted, or explanation-unstable predictions.

The present study addresses these requirements by evaluating a source-developed multi-class model under a strict unseen-domain protocol rather than by treating architectural complexity as the primary contribution. Class-specific calibration, predictive uncertainty, and class-conditional conformal prediction are evaluated as distinct reliability components. Demographic analyses are restricted to subgroup reliability for attributes supported by the available metadata. OOD separation and explanation stability are evaluated independently on DDI using a fixed checkpoint of the primary diagnostic architecture. Because independently verified lesion masks were unavailable, lesion-relevance scores were not computed. The selective-classification policy uses only source-fitted predictive-reliability quantities and does not incorporate DDI OOD or XAI outputs.

Prior works have contributed to multi-class representation learning, domain adaptation, fairness assessment, uncertainty quantification, and explainability, as shown in Table 1. However, these reliability dimensions remain methodologically distinct. Existing approaches either rely on target-domain data, measure fairness under limited demographic scenarios, estimate uncertainty without class-wise calibration, or measure explanation relevance without assessing the stability of attribution under acquisition shifts. None of the reviewed studies jointly evaluate unseen cross-device generalization, subgroup-specific calibration, predictive uncertainty, conformal prediction, explanation stability, and reliability-guided selective triage in a unified multi-class framework.

## 3. Methodology

### 3.1. Study Design and Data Governance Protocol

We designed the framework to assess whether multi-class skin-lesion predictions remain discriminative, calibrated, and explanation-stable under external dataset transfer, while separately auditing subgroup-specific reliability for demographic attributes supported by the available metadata. The subgroup analyses were not designed to establish demographic parity, fairness, or equity.

**Figure 2:**
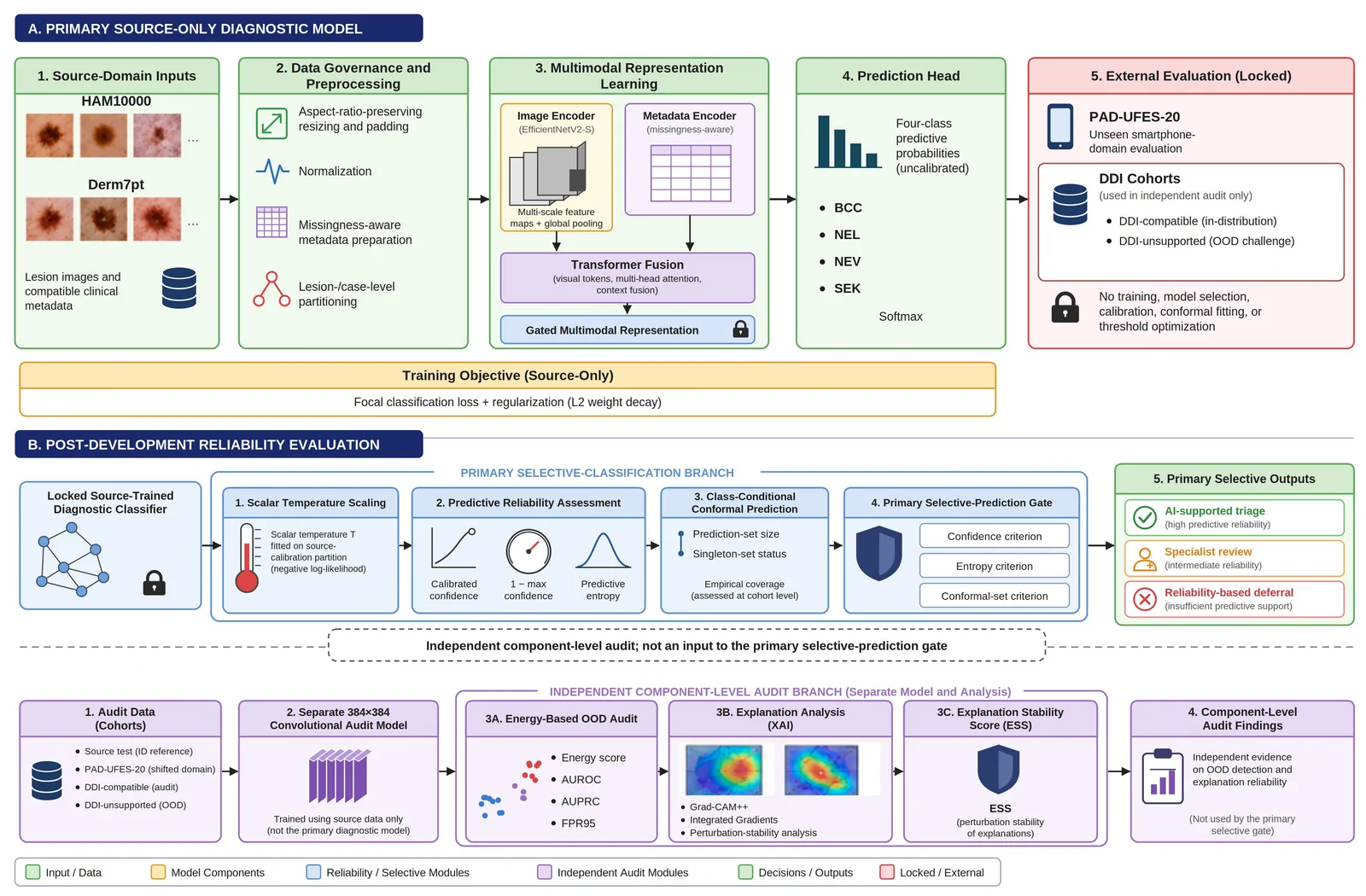
Methodological architecture of the primary selective-classification framework and independent component-level reliability audit. (**A**) Primary source-only diagnostic model; (**B**) Post-development reliability evaluation.

The methodological architecture of this study is shown in Figure 2, which distinguishes the primary source-only diagnostic pathway from the independent component-level reliability audit. Panel A shows the main diagnostic model. Images and compatible clinical metadata from HAM10000 and Derm7pt undergo standard source-domain preprocessing and lesion- or case-level partitioning. Multi-scale visual features are extracted using EfficientNetV2-S, which are combined with missingness-aware clinical metadata and transformer-based contextual fusion to generate uncalibrated four-class predictive probabilities for BCC, MEL, NEV, and BK. The PAD-UFES-20 set is completely locked during model development and only used for evaluation on unseen smartphone-domain data. The DDI dataset is only to be used for the independent post-development OOD and explanation audit. The two post-development branches in panel B differ methodologically. The primary selective-classification branch uses source-fitted class-specific probability calibration, calibrated confidence, predictive entropy, class-conditional conformal prediction, and a source-fitted selective-classification rule. The independent audit branch uses a fixed, prespecified Seed-17 checkpoint of the primary diagnostic architecture to evaluate DDI OOD separation and explain stability using Grad-CAM++ and Integrated Gradients. DDI OOD and XAI measurements are reported as independent audit evidence and are not used as inputs to the primary selective-classification gate. All calibration, conformal, and selective-decision parameters are fitted exclusively on predefined source-domain partitions. Cross-Device Multi-class Evaluation Task. HAM10000 and Derm7pt were treated as independent source domains, whereas PAD-UFES-20 was retained as a completely unseen smartphone-acquired target domain. Because the original datasets do not share an identical diagnostic taxonomy, the analysis was restricted to four harmonized study endpoints: basal cell carcinoma (BCC), melanoma (MEL), melanocytic nevus (NEV), and benign keratosis (BK). BK was defined as a dataset-harmonized study endpoint rather than as a claim of exact histopathological equivalence across datasets. HAM10000 BKL includes a broader benign-keratosis grouping, whereas the corresponding Derm7pt and PAD-UFES-20 categories are narrower seborrheic-keratosis labels. These categories were therefore mapped to the common BK endpoint for the present four-class evaluation.

Y = {BCC,MEL,NEV,BK}

All model-development procedures, including training, model selection, probability calibration, conformal fitting, and reliability-threshold selection, were performed exclusively using predefined source-domain partitions. PAD-UFES-20 was accessed only after all model parameters and source-fitting reliability settings had been fixed.

This study used only publicly released or research-accessible versions of HAM10000, Derm7pt, PAD-UFES-20, and DDI. HAM10000 was obtained from the Harvard Dataverse/ISIC release (DOI: 10.7910/DVN/DBW86T; accessed 29 September 2026). The dataset originates from dermoscopic collections acquired at the Medical University of Vienna, Austria, and the skin-cancer practice of Cliff Rosendahl in Queensland, Australia. The original dataset report documents ethical approval at the contributing institutions, expert technical review, ground-truth assignment based on histopathology, reflectance confocal microscopy, longitudinal follow-up, or expert consensus, and removal of potentially identifiable image content and EXIF information before public release. The dataset was used in accordance with the applicable public research-use terms.

Derm7pt (release_v0; accessed 29 September 2026) was obtained from the official Simon Fraser University Derm7pt distribution associated with Kawahara et al. The release contains paired clinical/dermoscopic images, diagnostic labels, seven-point checklist annotations, and structured metadata derived from the original expert-curated dermatology collection. Only the released research data and coded identifiers were used in the present study; No attempt at re-identification was made, and use was restricted to the non-commercial academic purposes permitted by the distribution terms.

PAD-UFES-20, Version 1, was obtained from Mendeley Data (DOI: 10.17632/zr7vgbcyr2.1, accessed 29 September 2026). The dataset was collected through the Dermatological and Surgical Assistance Program of the Federal University of Espírito Santo, Brazil. The original ethics dataset record reports approval by the university committee (No. 500002/478) and Plataforma Brasil (No. 4.007.097), collection under patient consent, and preservation of patient privacy. Diagnostic assessment involved experienced dermatologists; 58.4% of the lesions were biopsy-confirmed, including all skin-cancer cases. The public dataset is distributed under the CC BY 4.0 license.

The DDI (Diverse Dermatology Images) dataset was obtained from the Stanford AIMI/DDI research distribution (DOI: 10.71718/kqee-3z39; accessed 29 September 2026). The DDI dataset comprises retrospectively selected Stanford clinical images with pathology-confirmed diagnoses and expert dermatologic treatment. The released dataset underwent de-identification, including additional image cropping when required to protect privacy. Access and use were conducted in accordance with Stanford’s DDI Research Use Agreement, which limits the dataset to non-commercial research, prohibits re-identification, and restricts redistribution. Only the released de-identified research data were used in this study.

Formally, the restricted class space and the source-domain dataset structure are defined as follows:

(1) YB = {BCC, MEL, NEV, BK}

(2) $$D^{\left(d\right)}={\left\{(x_{i},m_{i},r_{i},y_{i},g_{i})\right\}}_{i=1}^{N_{d}},$$

where *x_i_* denotes an RGB lesion image, *m_i_* is the harmonized metadata vector, *r_i_* is a metadata-availability mask, *y_i_* is the diagnostic class, and *g_i_* is an eligible demographic group used during fairness-aware training.

Only variables with definitions compatible across the development datasets were retained. Age was standardized using statistics estimated solely from the source-training partition. Sex and anatomical site were encoded with fixed categorical mappings. Missing values were filled with source-training statistics and explicit availability indicators, so imputed values were not interpreted as observed clinical measurements. All partitions were made at the patient or lesion level. Images related to the same patient or lesion were assigned to only one partition. For each random seed, the source data were split into 60% for training, 10% for model-selection validation, 10% for calibration, 10% for reliability-threshold fitting, and 10% for internal testing. Partitions were stratified by source domain and diagnostic class. When the size of the group allowed, demographic stratification was employed. PAD-UFES-20 and DDI were not used for training, architecture selection, early stopping, probability calibration, conformal fitting, or reliability-threshold optimization. Subgroup reliability was evaluated using discrimination and calibration metrics for demographic attributes with sufficiently complete metadata.

The DDI dataset was retained exclusively for the independent post-development audit. Diagnoses outside the harmonized four-class label space formed the external OOD challenge, whereas diagnosis-compatible DDI cases were used only for the explanation-stability audit. DDI results did not influence model selection or any source-fitted reliability parameter. All images were resized to 224 × 224 pixels with aspect-ratio-preserving padding and normalized in the same manner across domains. Augmentation used for training included horizontal reflection, rotations of  ±20^∘^, moderate changes in brightness and contrast, gamma correction, slight Gaussian blur, controlled resolution reduction, and JPEG compression. Transformation ranges were limited to preserve lesion identity. No stochastic augmentation was used for validation, calibration, threshold fitting, test, or audit images.

### 3.2. Primary Source-Domain Diagnostic Classifier

We propose a model consisting of four components:

1. A multi-scale convolutional encoder;
2. A lightweight context module based on transformers;
3. A missingness-aware metadata encoder;
4. A gated multi-modal fusion classifier.

We use an ImageNet-initialized EfficientNetV2-S encoder to extract feature maps at three resolution levels. Each feature map is projected to a common embedding dimension, normalized in space, and converted into visual tokens. The resulting token sequence is then processed by two transformer encoder blocks with eight attention heads, a 256-dimensional embedding, a feed-forward expansion ratio of 4, and a dropout of 0.10. The contextual visual representation can be expressed compactly as

(3) *z_v_* = *T_ω_*([*P*_3_, *F*^(3)^, (*x*)*P*_4_*F*^(4)^(*x*)*P*_5_*F*^(5)^(*x*)]),

where *F*^(𝓁)^ denotes the convolutional feature map obtained at level 𝓁, *P*_𝓁_ is a learnable projection, and *T_ω_* is the lightweight transformer context encoder.

The metadata values and their availability indicators are jointly encoded:

(4) *z_m_* = *M_ξ_*(*m*‖*r*),

where *M_ξ_* is a two-layer multilayer perceptron.

Visual and metadata representations are integrated through a learned gate:

(5) *g* = *σ*(*W_g_*[*z_v_*‖*z_m_*] + *b_g_*),*z* = *g*⊙*W_v_z_v_* + (1 − *g*)⊙*W_m_z_m_*.

This mechanism allows the model to reduce its dependence on metadata when variables are missing or weakly informative.

The final class probabilities are

(6) $$p_{c}=\frac{\exp\left([W_{c}z+b_{c}{]}_{c}\right)}{\sum_{j=1}^{C}\exp([W_{c}z+b_{c}{]}_{j})}.$$

The learned visual representation is described as domain robust rather than domain invariant. The method aims to reduce acquisition-specific variation but does not assume that all domain information can be eliminated.

Controlled architectural ablation was performed across the same five prespecified seeds and source partitions used for the full model. Four variants were evaluated: (i) no transformer, in which transformerization was replaced by mean contextual aggregation of the projected multi-scale visual representation; (ii) no metadata, in which the clinical-metadata branch was removed; (iii) mean fusion, in which learned gated fusion was replaced by fixed mean fusion; and (iv) CNN-only, in which both transformer contextualization and metadata fusion were removed. Training, source partitions, optimization, and external evaluation procedures were otherwise held constant.

### 3.3. Imbalance-Aware Source-Domain Training

Training jointly addresses class imbalance, source-domain discrepancy, and demographic worst-group performance.

#### Imbalance-Aware Classification

The focal classification objective is

(7) $$L_{\mathrm{cls}}=-\frac{1}{|B|}\sum_{i\in B}\alpha_{y_{i}}{\left(1-p_{i,y_{i}}\right)}^{\gamma }\log\left(p_{i,y_{i}}+\varepsilon \right),$$

where ℬ is a mini-batch, *γ* controls the emphasis assigned to difficult examples, and $\alpha_{y_{i}}$ is calculated from the source-training class distribution.

Group-Aware Robustness Optimization. This group-aware optimization was intended to reduce sensitivity to represented demographic groups during source training and was not interpreted as evidence that demographic fairness or parity had been achieved. Sex is the main training-group attribute used when available. The age groups are considered as a secondary evaluation. Only intersectional groups meeting prespecified minimum sample requirements are included. empirical group risk for demographic group g is as follows:

(8) $$R_{g}=\frac{1}{|B_{g}|}\sum_{i\in B_{g}}l_{i},$$

where 𝓁*_i_* is the sample-level focal loss.

The implemented group-robustness term used a smooth approximation to the worst-group loss:

(9) $$L_{\mathrm{group}}=\tau \log_{\sum }\exp\left(\frac{R_{g}}{\tau }\right),\;\tau =0.10$$

The primary training objective was

(10) *L*_train_ = *L*_cls_ + 0.10*L*_group_.

Conditional source alignment was disabled in the primary five-seed configuration (*λ*_align_ = 0). Model parameters were optimized with AdamW using a weight decay of 1 × 10^−4^; no separate explicit *L*_2_ penalty was added to the training loss.

Where Θ contains all trainable model parameters. Group-risk estimates are used to prevent individual batches with missing or very small groups from dominating optimization.

### 3.4. Calibration, Uncertainty, and Conformal Prediction

For Source Only. All reliability parameters are fitted using source-domain partitions only.

#### 3.4.1. Class-Specific Temperature Scaling and Predictive Entropy

Class-specific temperature scaling was fitted exclusively to the designated source probability-calibration subset. A separate temperature parameter was estimated for each diagnostic class by minimizing multi-class negative log-likelihood. The fitted class-specific temperatures were then frozen and applied without modification to the internal source test sets and PAD-UFES-20. No external PAD-UFES-20 observations were used to estimate or select a temperature parameter for both the internal and external evaluation sets. The calibrated probability assigned to class *c* was computed.

Where *ε* > 0 is a small numerical constant used to avoid evaluating the logarithm at zero.

The conformal miscoverage level was fixed a priori at *α* = 0.10, corresponding to a nominal 90% coverage target. The value was not tuned on PAD-UFES-20.

#### 3.4.2. Predictive Uncertainty and Error Detection Measures

Predictive uncertainty was derived from the class probabilities after class-specific temperature scaling. Three complementary measures were evaluated as indicators of prediction error: one minus the maximum calibrated confidence, predictive entropy, and conformal prediction set size. Maximum confidence. One minus the maximum confidence was defined as

(11) $$U_{\mathrm{conf}}(x)=1-\max_{c}{\hat{p}}_{c}(x;T^{*}),$$

where ${\hat{p}}_{c}(x;T^{*})$ denotes the class-specifically calibrated probability assigned to class *c*. Predictive entropy was computed as

(12) $$U_{\mathrm{ent}}(x)=-\sum_{c=1}^{C}{\hat{p}}_{c}(x;T^{*})\log\left[{\hat{p}}_{c};(xT^{*})+\varepsilon \right],$$

where *ε* > 0 is a small numerical constant introduced to avoid evaluating the logarithm at zero. Conformal ambiguity was represented by the size of the prediction set,

(13) *U*_set_(*x*) = ∣𝒞*_α_*(*x*)∣,

where larger prediction sets indicate lower label specificity. These three quantities were evaluated independently for their ability to distinguish incorrect from correct predictions using AUROC and AUPRC. No Monte Carlo dropout or mutual-information-based epistemic uncertainty estimate was included in the reported analysis.

For calibration sample *i*, the nonconformity score is

(14) $$\begin{array}{cccc} & s_{i}=1-{\bar{p}}_{i,y_{i}}. & & \end{array}$$

Class-conditional quantiles are estimated separately for each source domain when the corresponding domain–class subset contains at least the prespecified minimum number of samples. Otherwise, a pooled class-specific quantile is used. The conservative source threshold is

(15) $${\hat{q}}_{c}^{\;\mathrm{rob}}=\max_{d}{\hat{q}}_{d,c}.$$

The conformal prediction set is

(16) Cα(x)={c:1−p‾c(x)≤q^c rob}.

The conformal model is fitted entirely on source data. Nominal finite-sample coverage is interpreted under the calibration assumptions. Coverage on PAD-UFES-20 is therefore evaluated empirically rather than presented as guaranteed under cross-device distribution shift.

### 3.5. OOD and Explanation Audit at Independent Component Level

Out-of-distribution detection and explanation stability were evaluated as an independent post-development audit and were not incorporated into the primary selective-classification gate. This secondary audit was included to characterize failure modes that are not directly measured by the supported-class cross-device evaluation on PAD-UFES-20. It was not designed as a second operational decision pathway. Specifically, the DDI dataset was used to assess whether the fixed primary architecture could distinguish diagnoses outside the supported four-class label space and whether its post hoc attribution patterns remained stable under prespecified diagnosis-preserving perturbations. The resulting OOD and XAI measurements were therefore treated as post-development evidence about the model and were not used to alter diagnostic predictions or selective-classification decisions. To remove the model mismatch present in the original implementation, the audit was repeated using a fixed prespecified Seed-17 checkpoint from the trained primary diagnostic architecture rather than a separately trained 384 × 384 convolutional model. No model parameter, probability-calibration parameter, conformal threshold, or selective-decision threshold was modified on the basis of DDI performance.

DDI remained completely excluded from diagnostic training, validation, early stopping, model selection, probability calibration, conformal fitting, and selective-threshold optimization. Diagnoses outside the supported four-class label space were used solely for post-development OOD assessment, whereas diagnosis-compatible DDI cases were used only for the explanation-stability audit. Consequently, the resulting OOD and XAI measurements constitute independent audit evidence and do not alter any prediction, acceptance decision, specialist-review assignment, or reliability-based deferral produced by the primary selective-classification pathway.

#### 3.5.1. Dual-Method Explanation Analysis

Generate Grad-CAM++ and Integrated Gradients for the predicted class. Attribution maps are corrected, scaled to input resolution, and normalized to unit attribution mass. The stability of the explanation was evaluated under six prespecified diagnosis-preserving perturbations: brightness factor 1.15, contrast factor 1.15, gamma 1.15, Gaussian blur with radius 1.0, JPEG compression at quality 70, and downsampling to one-half of the original linear resolution followed by resizing to the original dimensions. The stability score for explanation method m was defined as follows:

(17) $$\mathrm{ES}S_{m}(x)=\frac{1}{K}\sum_{k=1}^{K}\frac{\rho (A_{m},(x)A_{m}(x_{k}^{\prime }))+1}{2},$$

where *m* ∈ {GC^++^,IG} and *ρ* is Spearman rank correlation.

The combined Explanation Stability Score (ESS) was defined as the mean of the Grad-CAM++ and Integrated Gradients stability scores. ESS was used only as a post-development audit measure and was not an input to the selective-classification rule.

(18) $$\mathrm{ESS}(x)=\frac{\mathrm{ES}S_{GC^{++}}(x)+\mathrm{ES}S_{\mathrm{IG}}(x)}{2}.$$

Independently verified lesion masks were not available for the audited source, PAD-UFES-20, and DDI subsets. Lesion-relevance scores were therefore not computed in the reported analysis. Explanation stability is consequently interpreted only as robustness of the attribution pattern to the prespecified perturbations and not as evidence of lesion localization, diagnostic correctness, or causal validity:

(19) $$\mathrm{LRS}(x)=\frac{1}{2}\sum_{m}\frac{\sum_{u}A_{m}(u)M(u)}{\sum_{u}A_{m}(u)+\varepsilon }$$

This prevents the operational framework from depending on manual lesion masks that may not be available during deployment.

#### 3.5.2. OOD Detection

OOD detection is evaluated independently from predictive uncertainty and conformal-set size. The energy score is

(20) $$\begin{array}{cccc} & E(x)=-\tau_{E}\log\sum_{c=1}^{C}\exp(\frac{{\overset{-}{o}}_{c}}{\tau_{E}}). & & \end{array}$$

OOD detection was evaluated only as an independent post-development ranking problem. The fixed Seed-17 internal source-test set was treated as in-distribution data, and the 362 unsupported DDI diagnoses formed the external OOD challenge. One minus maximum softmax probability, predictive entropy, and energy score were compared using AUROC, AUPRC, and FPR95. No OOD decision threshold was used in the primary selective-classification pathway, and the DDI dataset was not used to fit or modify any selective-decision threshold.

### 3.6. Selective Triage and Reliability Evaluation

#### 3.6.1. Source-Fitted Reliability Gate

The primary selective-classification gate was fitted exclusively using the predefined source reliability-threshold partition after diagnostic training, probability calibration, and conformal fitting had been completed. PAD-UFES-20 and DDI were not used to select, optimize, or modify any selective-decision threshold.

The implemented gate used only reliability quantities derived from the frozen primary diagnostic model and the source-fitted calibration/conformal pipeline: one minus maximum calibrated confidence, predictive entropy, logit-derived energy, and conformal prediction-set size. These signals were standardized using parameters estimated from the source reliability-threshold partition and combined into a source-fitted reliability-risk score.

The logit-derived energy term used here should not be interpreted as the DDI OOD detector reported in Section 3.5. It is calculated directly from the primary model logits and is used only as a source-derived reliability feature. By contrast, DDI-based OOD separation is evaluated independently as a post-development audit. Neither DDI OOD performance nor Grad-CAM++/Integrated Gradients explanation-stability measurements are inputs to the selective-classification gate.

(21) *R*(*x*) = 0.30*r*~*conf* (*x*) + 0.20*r*~*ent* (*x*) + 0.20*r*~*MI* (*x*) + 0.15*r*~*energy* (*x*) + 0.15*r*~*confset* (*x*),

where ${\tilde{r}}_{\mathrm{MI}}\left(x\right)=0$ in the reported deterministic evaluation. All standardization parameters were estimated from the source reliability-threshold partition and frozen before external evaluation. The energy term in the composite reliability-risk score above is a source-derived logit-risk feature and is distinct from the independent DDI OOD audit reported in Section 3.5. is a source-derived logit-risk feature and is distinct from the independent DDI OOD audit reported in Section 3.5. The software implementation retains a mutual-information field for compatibility with stochastic-inference configurations; however, the reported evaluation used a single deterministic forward pass (mc_passes = 1). Mutual information was therefore identically zero and made no sample-specific contribution to the executed reliability score. It is not interpreted as an epistemic uncertainty estimate in this study.

#### 3.6.2. Three-Level Decision Policy

Two source-derived threshold decisions were applied to the composite reliability-risk score. Predictions below the acceptance threshold were retained for AI-supported triage, predictions between the acceptance and review thresholds were assigned to specialist review, and predictions above the review threshold were assigned to reliability-based deferral. Only cases in the retained state contributed to accepted-case accuracy. The policy was evaluated as an experimental selective-classification mechanism and was not interpreted as an autonomous clinical decision system.

The framework returns one of three actions:

(22) $$A\left(x\right)=\left\{\begin{array}{cc} \mathrm{AI}-\mathrm{supported triage}, & R\left(x\right)\leq \tau_{\mathrm{acc}},\\ \mathrm{Specialist review}, & \tau_{\mathrm{acc}}<R\left(x\right)\leq \tau_{\mathrm{rev}},\\ \mathrm{Reliability}-\mathrm{based deferral}, & R\left(x\right)>\tau_{\mathrm{rev}}. \end{array}\right.$$

For accepted predictions, the predicted lesion class is mapped to a prespecified experimental triage-priority category. The mapping is fixed before external testing and is not interpreted as an autonomous clinical decision. The framework is intended to support prioritization and specialist review rather than replace dermatological assessment.

#### 3.6.3. Threshold Optimization

The acceptance and review thresholds were estimated exclusively from the source reliability-threshold partition and frozen before external evaluation. Threshold selection was designed to preserve high accuracy among retained cases while satisfying the prespecified source-domain coverage requirement. Selective performance was summarized using retained coverage, accepted-case accuracy, selective risk, specialist-review assignment, and reliability-based deferral. Neither PAD-UFES-20 nor DDI results were used to modify the thresholds.

Thresholds are selected by minimizing selective classification risk while preserving a minimum retained coverage:

(23) $$\tau^{*}=\arg\min_{\tau }[R_{\mathrm{sel}}(\tau )+\lambda_{\mathrm{def}}(1-\mathrm{Cov}(\tau ))],\mathrm{Cov}(\tau )\geq \kappa_{\min}.$$

Threshold fitting is completed before any external test result is examined.

Threshold-sensitivity analysis was performed after fixing the primary source-only procedure. The target retained-coverage level used during source threshold fitting was varied across prespecified operating points of 25%, 35%, 45%, 55%, and 65%. For each operating point, the acceptance threshold was re-estimated using only the source reliability-threshold partition, while PAD-UFES-20 remained completely excluded from threshold fitting. The resulting retained coverage, accepted-case accuracy, selective risk, and deferral behavior were then evaluated post hoc on both the source test set and PAD-UFES-20. This analysis was intended to characterize the coverage–risk trade-off rather than to select an operating point using external data.

**Figure 3:**
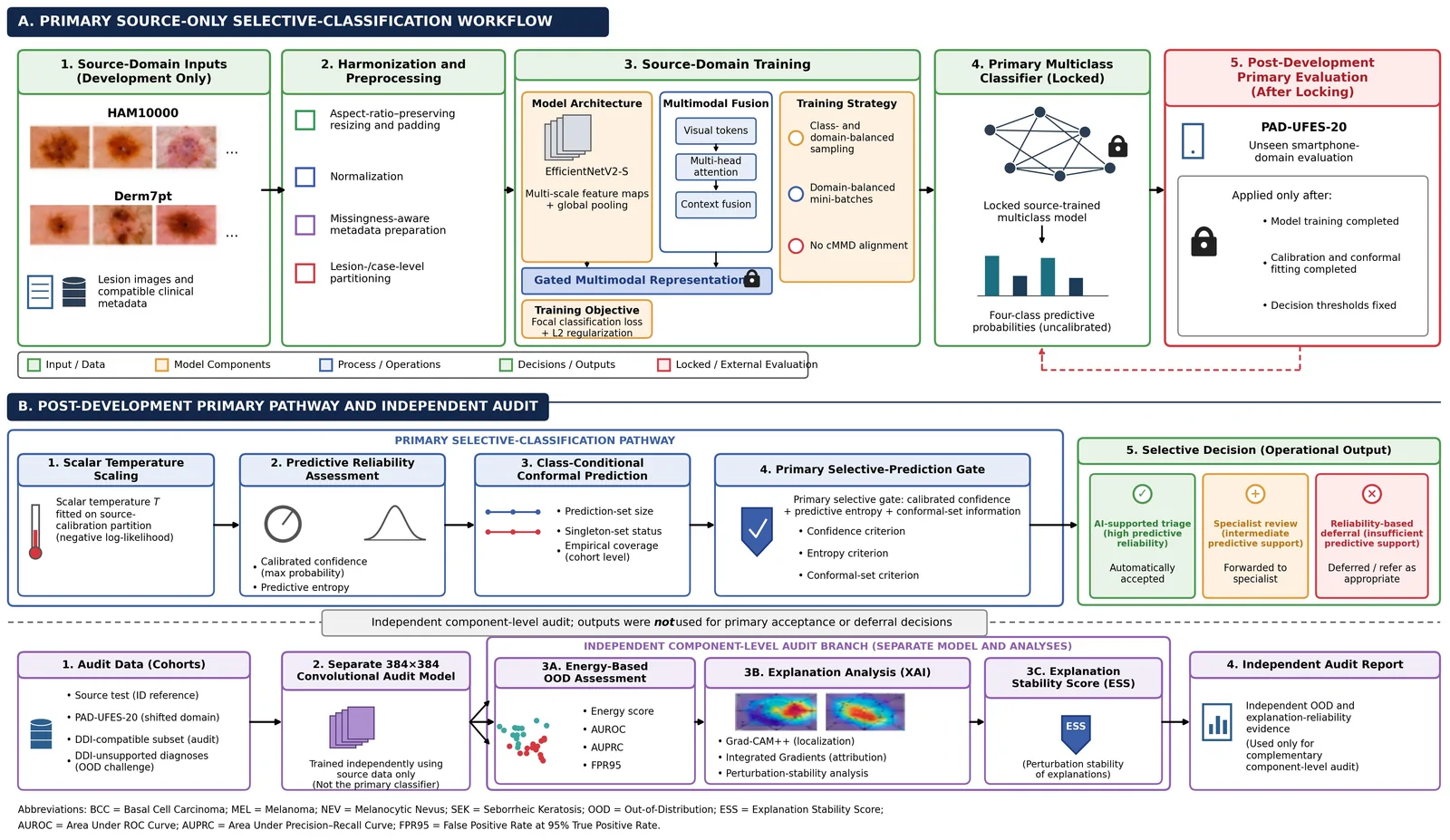
Source-only selective-classification workflow with an independent post-development DDI audit. (**A**) Primary source-only selective-classification workflow; (**B**) Post-development primary pathway and independent audit.

Figure 3 separates the post-development evaluation into two methodologically distinct pathways. The primary selective-classification pathway uses only source-fitted predictive-reliability signals derived from the frozen diagnostic model, probability calibration, and conformal prediction. The independent audit pathway evaluates OOD separation and explanation stability after model development. PAD-UFES-20 and DDI remain excluded from all training and source-threshold fitting procedures. Importantly, the outputs of the independent DDI OOD/XAI audit are reported as supplementary reliability evidence and are not fed back into the selective-classification gate.

The primary pipeline uses HAM10000 and Derm7pt for model development, source-fitted class-specific probability calibration, predictive confidence/entropy, class-conditional conformal prediction, and selective classification. PAD-UFES-20 is excluded from all development and threshold-fitting procedures and is used only for unseen external evaluation. The independent DDI audit uses a fixed prespecified Seed-17 checkpoint of the primary diagnostic architecture for OOD assessment and Grad-CAM++/Integrated Gradients explanation-stability analysis. DDI OOD/XAI outputs do not modify the primary selective-classification decisions.

### 3.7. Algorithmic Implementation and Reproducibility

The following algorithms summarize the order of implementation of source-domain training, reliability fitting, independent auditing, and selective decision support. The individual components are established methods; the contribution is their source-restricted evaluation protocol and reliability-oriented integration. Software environment. All experiments were implemented in Python 3.12 in a GPU-enabled Kaggle environment. Model development and inference were performed using PyTorch 2.14.0 and torchvision 0.29.0. Numerical processing, data handling, statistical analysis, and performance evaluation were conducted using NumPy 2.5.2, pandas 2.3.3, SciPy 1.18.1, and scikit-learn 1.9.0. Pillow 12.3.0 was used for image processing, PyYAML 6.0.3 for configuration management, and Matplotlib 3.11.1 for scientific visualization. Captum 0.9.0 was used for attribution-based explainability where applicable. 

The source-only multimodal training procedure is summarized in Algorithm 1. The subsequent source-only reliability fitting and external evaluation workflow is presented in Algorithm 2, while the source-fitted reliability-gated selective-classification procedure is described in Algorithm 3.
**Algorithm 1:** Source-Only Group-Aware Multimodal Training**Input**:  HAM10000 source domain D1  Derm7pt source domain D2  Harmonized class set Y  Model parameters Θ   E**Output:**    Source-validated model Θ***Method:** 1: Harmonize diagnostic labels and metadata across D1 and D2 2: Construct patient-level train, validation, calibration, threshold-fitting, and internal-test partitions 3: Initialize model parameters Θ 4: for epoch = 1 to E do 5: Construct domain-balanced and group-aware mini-batches 6: for each mini-batch B do 7: Extract multi-scale visual features 8: Generate transformer contextual representation zv 9: Encode metadata and availability indicators zm 10: Compute gated multimodal representation z 11: Compute class probabilities and focal loss Lcls 12: Compute the mean focal loss *R_g_* for each sufficiently represented supported group. 13: Compute the smooth worst-group robustness term *L*_group_ with *τ* = 0.10. 14: Compute *L*_train_ = *L*_cls_ + 0.10*L*_group_. 15: Update model parameters using AdamW with weight decay 1 × 10^−4^. 16: end for 17: Evaluate macro-F1, worst-group F1, and validation loss 18: Apply early stopping using source validation only 19: end for 20: Return the best source-validated model Θ*


**Algorithm 2:** Source-Only Reliability Fitting and External Evaluation**Input:** Trained primary diagnostic checkpoint; probability-calibration subset; conformal-calibration subset; source reliability-threshold partition; source test set; PAD-UFES-20.**Output:** Calibrated probabilities, conformal prediction sets, source-fitted selective decisions, and external reliability measurements.**Method:**Freeze the trained primary diagnostic checkpoint.Fit probability calibration using only the designated source calibration subset.Apply the fixed calibration parameters to the independent conformal-calibration subset.Estimate class-conditional conformal thresholds using this subset only.Apply the fixed calibration and conformal pipeline to the source reliability-threshold partition.Compute calibrated-confidence risk, predictive entropy, logit-derived energy, and conformal-set size.Fit the source selective-classification rule and freeze all scaling parameters and decision thresholds.Apply the complete fixed pipeline to the source test set and PAD-UFES-20 without adaptation.Report discrimination, calibration, conformal, uncertainty-based error-detection, and selective-classification performance.Perform DDI OOD and explanation-stability analyses separately using the prespecified fixed audit checkpoint.**Constraint:** PAD-UFES-20 and DDI results are not used to modify the diagnostic model, calibration parameters, conformal predictor, or selective-classification thresholds.
**Algorithm 3:** Source-Fitted Reliability-Gated Selective Classification**Input:** Calibrated probabilities; predictive entropy; logit-derived energy; conformal prediction set; source-fitted standardization parameters; signal weights; acceptance threshold; review threshold.**Output:** AI-supported triage, specialist review, or reliability-based deferral.**Method:**Compute one minus maximum calibrated confidence.Compute predictive entropy.Compute the logit-derived energy reliability feature.Determine conformal prediction-set size.Apply the fixed source-derived standardization to the four reliability signals.Combine the standardized signals to obtain the reliability-risk score.If the score is below the acceptance threshold, retain the prediction for AI-supported triage.If the score lies between the acceptance and review thresholds, assign the case to specialist review.Otherwise, assign the case to reliability-based deferral.Report retained coverage, accepted-case accuracy, selective risk, and non-retained rate.DDI OOD scores and explanation-stability measurements are not inputs to this decision rule.


#### Sensitivity Analysis of Exploratory Conditional Alignment

The conditional source alignment was not part of the primary five-seed diagnostic analysis. It was evaluated separately as an exploratory single-seed sensitivity analysis to examine whether diagnosis-conditional source alignment may reduce majority-class dominance under external transfer. For this analysis, a class-conditional multi-kernel maximum mean discrepancy term was added to the diagnostic objective. As the experiment was run with a single random seed and was not used for model selection, results were considered hypothesis-generating and not interpreted as confirmatory evidence or evidence of superiority. Marginal alignment may mistakenly pull samples from different diagnostic classes into the same representation. Therefore, source domains were conditionally aligned on the diagnosis. For class C, the meaning embedding in the source domain is as follows:

(24) $$\mu_{d,c}=\frac{1}{|B_{d,c}|}\sum_{i\in B_{d,c}}\Phi (z_{v,i}),$$

where Φ(⋅) is induced by a multi-kernel radial-basis function.

The class-conditional alignment loss is

(25) $$L_{\mathrm{cMMD}}=\sum_{c=1}^{C}\omega_{c}{\left\|\mu_{1,c}-\mu_{2,c}\right\|}_{H}^{2}.$$

Classes contribute to the respective update only if they are present in both source domain components of a mini-batch. We adopt domain-balanced mini-batches to alleviate unstable alignment.

We clarify that generative AI assistance was used exclusively as an author-support tool for language editing and linguistic refinement. It was not involved in data analysis, generation of results, or scientific decision-making.

## 4. Results

### 4.1. Experimental Setting and Data Integrity

The experimental evaluation was conducted in accordance with a strict source-only development protocol. HAM10000 and Derm7pt were taken as independent source domains, and PAD-UFES-20 was kept as a fully unseen smartphone-acquired target domain.

We only applied DDI for independent out-of-distribution and explanation-reliability auditing after the model was developed. The cross-device analysis was limited to four diagnostically compatible categories, namely basal cell carcinoma (BCC), melanoma (MEL), melanocytic nevus (NEV), and harmonized benign-keratosis endpoint (BK). The evaluated source cohort comprised 11,259 eligible images: 9431 from HAM10000 and 1828 from Derm7pt. The external PAD-UFES-20 cohort comprised 1376 eligible smartphone-acquired images. All source partitions were generated at the lesion- or clinical-case level. Therefore, related observations were prevented from appearing in different experimental subsets. The source data were split into training, model-selection, calibration, reliability-threshold, and internal-test sets. PAD-UFES-20 and DDI did not participate in any training, early stopping, probability calibration, conformal fitting, or threshold selection as shown in Table 2.

**Table 2:** Dataset composition and experimental role.

Dataset	BCC	MEL	NEV	BK	Total
HAM10000	514	1113	6705	1099	9431
Derm7pt	84	504	1150	90	1828
Combined source	598	1617	7855	1189	11,259
PAD-UFES-20	845	52	244	235	1376

The source data were highly imbalanced, with NEV accounting for 70.2% of the source cohort. The diagnostic distribution of PAD-UFES-20 was distinct, with BCC accounting for 61.4% of the external cohort. Therefore, macro-F1 was considered the primary discrimination metric, and accuracy, balanced accuracy, weighted-F1, Matthews correlation coefficient (MCC), and macro one-vs-rest AUROC were considered secondary outcomes.

**Figure 4:**
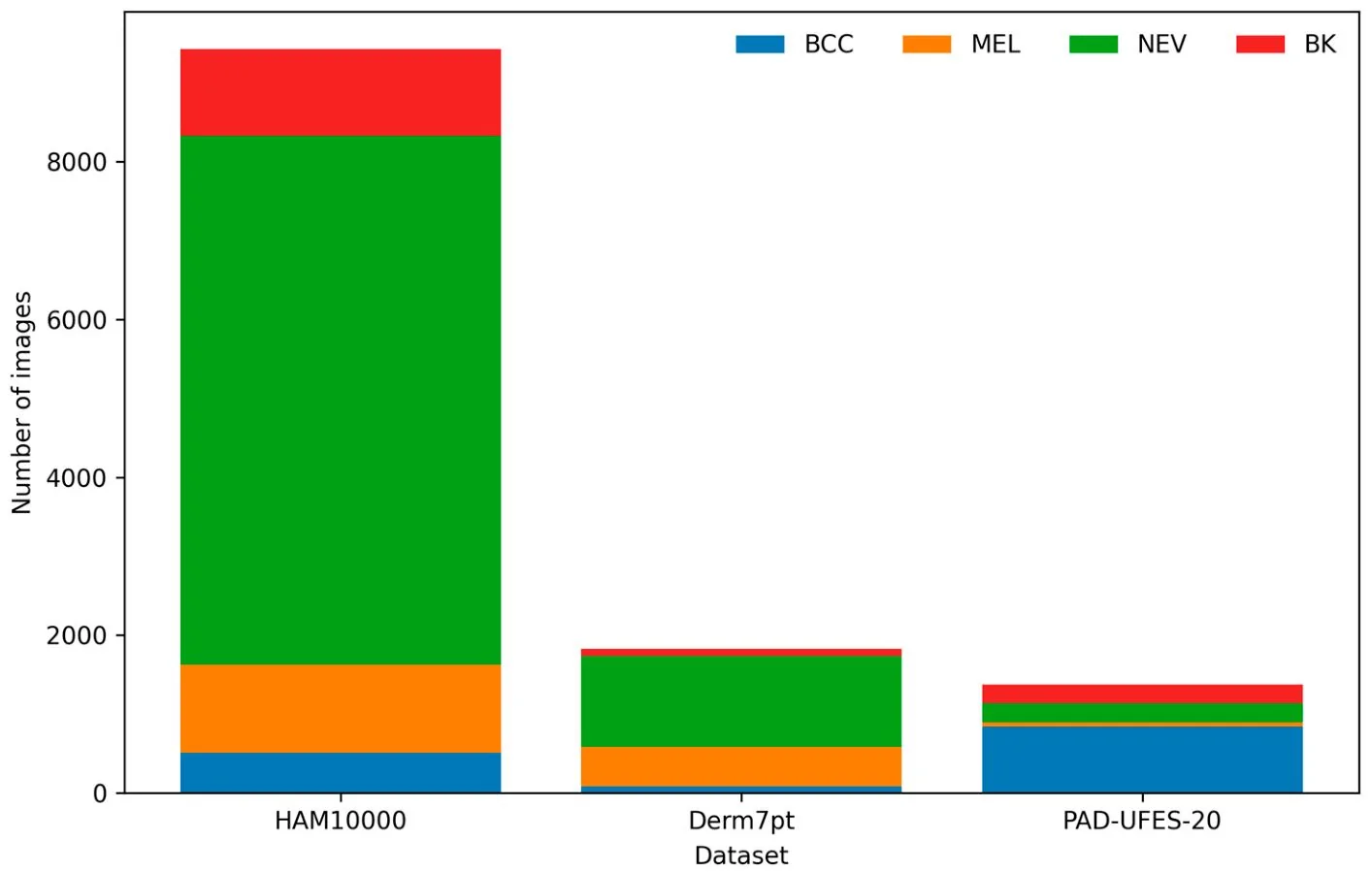
Diagnostic class distribution across HAM10000, Derm7pt, and PAD-UFES-20 after harmonization to the four study endpoints BCC, MEL, NEV, and BK.

Figure 4 shows marked differences in diagnostic distributions between the source and target datasets, as shown in Figure 4. NEV accounted for the majority of the source cohort, while BCC was the most common category in PAD-UFES-20. This change in distribution led to the joint interpretation of macro-F1, balanced accuracy, and MCC, rather than only overall accuracy.

### 4.2. Source-Domain Discrimination and Reproducibility

Across the five prespecified seeds, the fully uncalibrated diagnostic model achieved 82.25 ± 1.76% source-domain accuracy, 76.49 ± 1.67% balanced accuracy, a macro-F1 score of 74.27 ± 1.36%, a weighted-F1 score of 82.91 ± 1.50%, an MCC of 0.645 ± 0.028, and a macro one-vs-rest AUROC of 0.926 ± 0.005. Variation across seeds was modest for these aggregate discrimination measures as shown in Table 3.

**Table 3:** Aggregate discrimination across source and unseen smartphone domains.

Metric	Source	PAD-UFES-20	Change
Accuracy (%)	82.25 ± 1.76	65.96 ± 3.91	−16.29
Balanced accuracy (%)	76.49 ± 1.67	61.88 ± 1.31	−14.62
Macro-F1 (%)	74.27 ± 1.36	54.92 ± 2.60	−19.35
Weighted-F1 (%)	82.91 ± 1.50	68.53 ± 3.43	−14.38
MCC	0.645 ± 0.028	0.506 ± 0.040	−0.139
Macro-AUROC	0.926 ± 0.005	0.880 ± 0.011	−0.046

The diagnostic class distributions differed markedly between the development and external cohorts. In the combined source cohort, NEV accounted for 7855 of 11,259 images (69.8%), whereas BCC represented 598 images (5.3%). In PAD-UFES-20, by contrast, BCC accounted for 845 of 1376 images (61.4%), while NEV represented 244 images (17.7%). MEL prevalence also decreased from 14.4% in the source cohort to 3.8% externally. Consequently, the source-to-PAD-UFES-20 comparison involves both a change in image-acquisition conditions and a substantial diagnostic-prevalence shift. External performance differences are therefore not attributed to hardware acquisition alone as shown in Table 4.

**Table 4:** Class-wise source-domain performance.

Class	Precision	Sensitivity	Specificity	F1	AUROC
BCC	74.45 ± 4.62	78.88 ± 4.30	98.58 ± 0.33	76.47 ± 2.70	0.978 ± 0.014
MEL	51.81 ± 3.45	66.66 ± 4.20	90.16 ± 1.76	58.21 ± 2.66	0.862 ± 0.014
NEV	92.76 ± 0.86	86.77 ± 2.77	83.62 ± 2.61	89.65 ± 1.51	0.931 ± 0.006
BK	72.10 ± 4.80	73.67 ± 3.83	96.52 ± 0.76	72.76 ± 2.97	0.933 ± 0.008

The difference between weighted-F1 and macro-F1 reflects the strong influence of the dominant NEV class in the source cohort. Accordingly, overall accuracy and weighted-F1 were not interpreted as measures of balanced multi-class performance. Macro-F1, balanced accuracy, MCC, macro-AUROC, and class-specific measures were therefore reported jointly. Among these metrics, balanced accuracy and class-wise sensitivity provide information that is less directly driven by the observed class frequencies, whereas precision, F1-score, and MCC may still be affected by changes in prevalence and error composition.

### 4.3. External Generalization Under Acquisition and Class-Prevalence Shift

**Figure 5:**
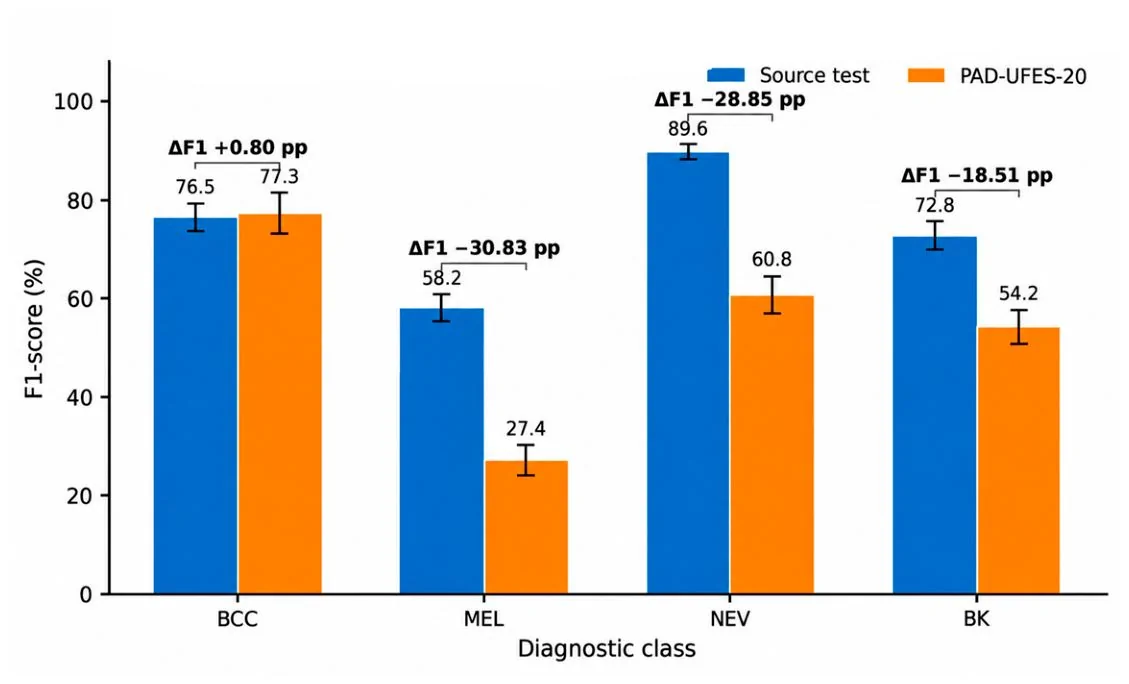
Class-specific F1-score comparison between the source test sets and PAD-UFES-20 across the five prespecified seeds.

External evaluation on PAD-UFES-20 assessed performance under a compound cross-dataset shift. PAD-UFES-20 differed from the development data not only in image acquisition, including smartphone-based clinical photography, but also substantially in diagnostic prevalence. The source cohort was predominantly composed of NEV, whereas PAD-UFES-20 was predominantly composed of BCC. The resulting performance changes should therefore be interpreted as external-transfer effects under combined acquisition and prevalence shifts rather than as an isolated estimate of device-induced degradation. Across the five prespecified seeds, external accuracy was 65.96 ± 3.91%, balanced accuracy was 61.88 ± 1.31%, macro-F1 was 54.92 ± 2.60%, weighted-F1 was 68.53 ± 3.43%, MCC was 0.506 ± 0.040, and macro one-vs-rest AUROC was 0.880 ± 0.011. Relative to the source-domain test sets, balanced accuracy decreased by 14.62 percentage points and macro-F1 by 19.35 percentage points, while macro-AUROC decreased by 0.046 as shown in Figure 5. Because these metrics have different sensitivities to class prevalence and decision thresholds, the external change was not interpreted from any single aggregate measure.

Bars show mean F1-score, and error bars show standard deviations across seeds 17, 29, 41, 53, and 71. The source-to-external change was +0.80 percentage points for BCC, −30.83 for MEL, −28.85 for NEV, and −18.51 for BK. The pattern demonstrates heterogeneous class-specific transfer under the combined acquisition, prevalence, and dataset-composition shift represented by PAD-UFES-20 and should not be interpreted as an isolated device effect.

External transfer was strongly class-dependent. As shown in Table 5 relative to the source-domain test sets, mean F1 changed from 76.47 ± 2.70% to 77.27 ± 4.02% for BCC (+0.80 percentage points), from 58.21 ± 2.66% to 27.38 ± 2.96% for MEL (−30.83 points), from 89.65 ± 1.51% to 60.80 ± 3.66% for NEV (−28.85 points), and from 72.76 ± 2.97% to 54.25 ± 3.33% for BK (−18.51 points). MEL was therefore the class most affected in terms of F1 degradation, followed closely by NEV. For MEL, both precision and sensitivity decreased markedly, whereas the reduction in NEV F1 was driven primarily by a large decrease in precision despite comparatively preserved sensitivity. BCC exhibited a different pattern: sensitivity decreased from 78.88% to 64.66%, but the accompanying increase in precision to 96.34% resulted in a largely preserved F1-score. The row-normalized confusion matrices further showed that 42.69 ± 6.29% of MEL cases were assigned to NEV and that 22.72 ± 4.05% of BK cases were also assigned to NEV. These findings demonstrate that the effect of external transfer was heterogeneous across diagnostic classes rather than a uniform deterioration of the classifier.

**Table 5:** Class-wise performance on unseen PAD-UFES-20 images.

Class	Precision	Sensitivity	Specificity	F1	AUROC
BCC	96.34 ± 0.44	64.66 ± 5.67	96.08 ± 0.62	77.27 ± 4.02	0.921 ± 0.016
MEL	21.03 ± 3.73	41.15 ± 5.86	93.52 ± 2.74	27.38 ± 2.96	0.863 ± 0.029
NEV	51.01 ± 5.37	75.74 ± 2.04	84.01 ± 3.56	60.80 ± 3.66	0.888 ± 0.022
BK	46.13 ± 2.25	65.96 ± 5.84	84.15 ± 0.98	54.25 ± 3.33	0.848 ± 0.020

**Figure 6:**
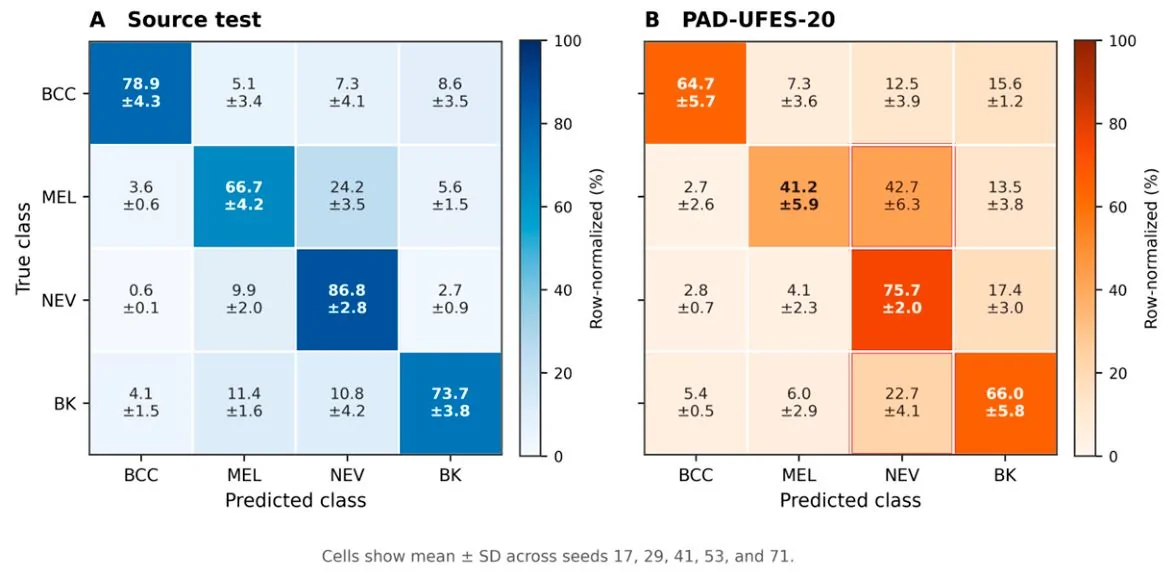
Row-normalized confusion matrices for the source test set and PAD-UFES-20 across the five prespecified seeds. (**A**) Source test; (**B**) PAD-UFES-20.

The Figure 6 explain each cell reports the mean percentage ± standard deviation of samples from a true diagnostic class assigned to each predicted class across seeds 17, 29, 41, 53, and 71. The diagonal cells represent class-specific sensitivity. The highlighted external off-diagonal cells identify the two dominant confusion pathways discussed in the text: MEL to NEV (42.69 ± 6.29%) and BK to NEV (22.72 ± 4.05%). Row normalization facilitates class-wise comparison despite the marked difference in diagnostic prevalence between the source and external cohorts.

#### Class-Prior Sensitivity Analysis

To assess whether the source-to-PAD-UFES-20 performance difference could be explained primarily by the marked difference in diagnostic prevalence, an additional class-balanced sensitivity analysis was performed without retraining, recalibration, or model adaptation. For each of the five primary seeds, equal numbers of images were sampled from each of the four diagnostic classes in both the source test set and PAD-UFES-20. The smallest PAD-UFES-20 class contains 52 images. Therefore, 52 images per class were used, producing 208 images per domain in each balanced replicate, producing 208 images per domain in each balanced replicate. The procedure was repeated 10,000 times using the fixed model predictions.

After equalizing the class distribution, source macro-F1 was 76.55 ± 1.66%, whereas PAD-UFES-20 macro-F1 was 61.74 ± 1.01%. The resulting gap was approximately 14.81 percentage points, compared with a 19.35-point gap in the full cohorts. MCC remained lower externally (0.504 ± 0.017) than internally (0.691 ± 0.022). These results indicate that diagnostic-prevalence shift explains part, but not all, of the observed external degradation. The remaining difference should not be interpreted as an isolated device effect, because acquisition conditions and other dataset-specific factors also differ between the cohorts.

### 4.4. Probability Calibration and Detection of Uncertainty-Based Errors

Class-specific temperature scaling was fitted exclusively to the source probability-calibration subsets. Across the five primary seeds, source ECE decreased from 6.14 ± 1.81% to 3.38 ± 0.38%, while PAD-UFES-20 ECE decreased from 21.11 ± 2.52% to 11.63 ± 3.80%. The corresponding changes in macro-AUROC were small and not uniformly favorable, emphasizing that improved probability calibration should not be interpreted as improved discrimination.

The class-specific results demonstrate that external degradation was heterogeneous rather than uniform across diagnoses. BCC maintained the highest external F1-score (77.27 ± 4.02%) and AUROC (0.921 ± 0.016), whereas MEL showed the largest reduction in decision performance, with an F1-score of 27.38 ± 2.96% despite an AUROC of 0.863 ± 0.029. NEV and BK showed intermediate external performance. The divergence between class-specific F1-score and AUROC, particularly for MEL, indicates that deterioration in the final multi-class decisions cannot be equated with complete loss of class-ranking information. At the same time, because the class priors differ substantially between source and PAD-UFES-20, these class-specific findings should not be interpreted as isolating an acquisition-device effect. They instead characterize how each diagnostic class behaved under the combined external shift as shown in Table 6.

**Table 6:** Calibration before and after class-specific temperature scaling.

Domain	Calibration	NLL	Brier	ECE (%)	Macro-AUROC
Source	Uncalibrated	0.5164 ± 0.0218	0.2621 ± 0.0174	6.14 ± 1.81	0.926 ± 0.005
Source	Class-specific TS	0.4309 ± 0.0186	0.2265 ± 0.0094	3.38 ± 0.38	0.936 ± 0.005
PAD	Uncalibrated	1.4896 ± 0.1925	0.5456 ± 0.0549	21.11 ± 2.52	0.880 ± 0.011
PAD	Class-specific TS	1.1581 ± 0.1492	0.5416 ± 0.0682	11.63 ± 3.80	0.870 ± 0.013

These findings indicate that uncertainty signals that discriminated between correct and incorrect source-domain predictions did not transfer reliably to the unseen smartphone domain as shown in Table 7.

**Table 7:** Uncertainty-based error detection.

Domain	Measure	AUROC	AUPRC
Source	1 − maximum calibrated confidence	0.818 ± 0.012	0.444 ± 0.041
Source	Predictive entropy	0.821 ± 0.008	0.456 ± 0.031
Source	Conformal-set size	0.619 ± 0.065	0.213 ± 0.049
PAD	1 − maximum calibrated confidence	0.695 ± 0.041	0.558 ± 0.051
PAD	Predictive entropy	0.685 ± 0.042	0.550 ± 0.044
PAD	Conformal-set size	0.560 ± 0.077	0.433 ± 0.083

**Figure 7:**
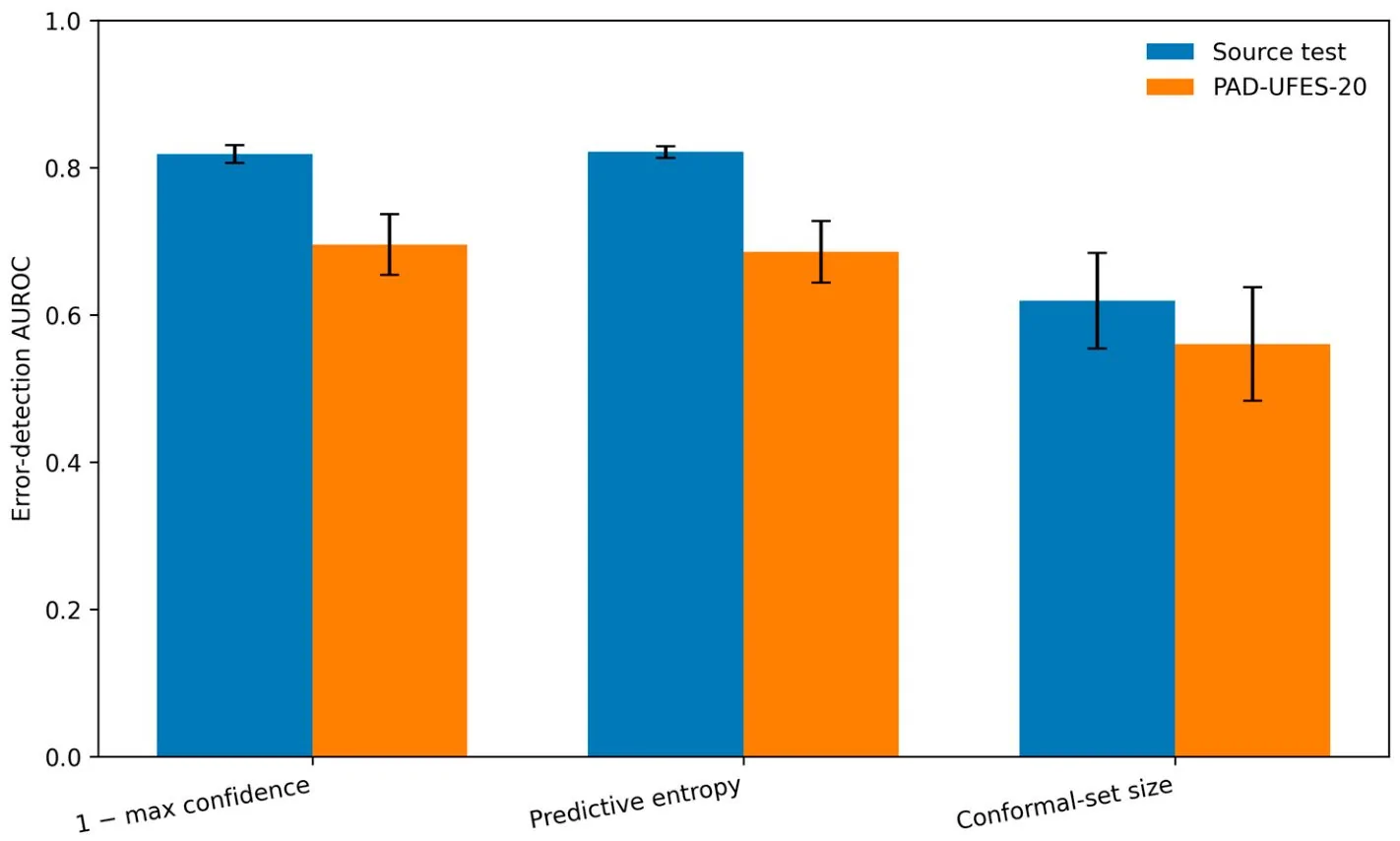
Transferability of uncertainty-based error detection from the source domain to PAD-UFES-20. Bars show mean AUROC across the five primary seeds, with error bars representing standard deviations.

As seen in Figure 7, class-specific temperature scaling provided a consistent calibration benefit across domains. Although source-domain ECE remained low, uncertainty-based error detection performed much worse after transfer to PAD-UFES-20. Confidence- and entropy-based error detection remained above chance externally but were substantially weaker than in the source domain, whereas conformal-set size approached chance-level discrimination. These results indicate reduced transferability of source-derived error-detection signals under external shift.

### 4.5. Selective Decision Support and Conformal Prediction

Class-conditional conformal prediction was fitted exclusively to the source conformal-calibration subsets at a prespecified nominal coverage of 90%. Across the five primary seeds, empirical source-test coverage was 90.39 ± 1.18%, with a mean prediction-set size of 1.56 ± 0.04 and a singleton-set rate of 50.96 ± 5.73%. On PAD-UFES-20, empirical coverage decreased to 84.24 ± 5.21%, mean prediction-set size increased to 1.89 ± 0.11, and the singleton-set rate decreased to 26.35 ± 3.81%.

Table 8 explain the source-fitted selective policy retained 32.37 ± 3.45% of source-test cases with an accepted-case accuracy of 97.86 ± 1.04%. On PAD-UFES-20, retained coverage decreased to 11.50 ± 4.53%, while accepted-case accuracy was 92.97 ± 3.64%. The corresponding non-retained rates were 67.63 ± 3.45% and 88.50 ± 4.53%, respectively. Non-retained cases include both specialist-review and reliability-based deferral states and therefore should not be interpreted as deferral alone.

**Table 8:** Conformal prediction and selective triage.

Domain	Coverage	Mean Set Size	Singleton	Retained	Accepted Accuracy	Non-Retained
Source	90.39 ± 1.18	1.56 ± 0.04	50.96 ± 5.73	32.37 ± 3.45	97.86 ± 1.04	67.63 ± 3.45
PAD	84.24 ± 5.21	1.89 ± 0.11	26.35 ± 3.81	11.50 ± 4.53	92.97 ± 3.64	88.50 ± 4.53

**Figure 8:**
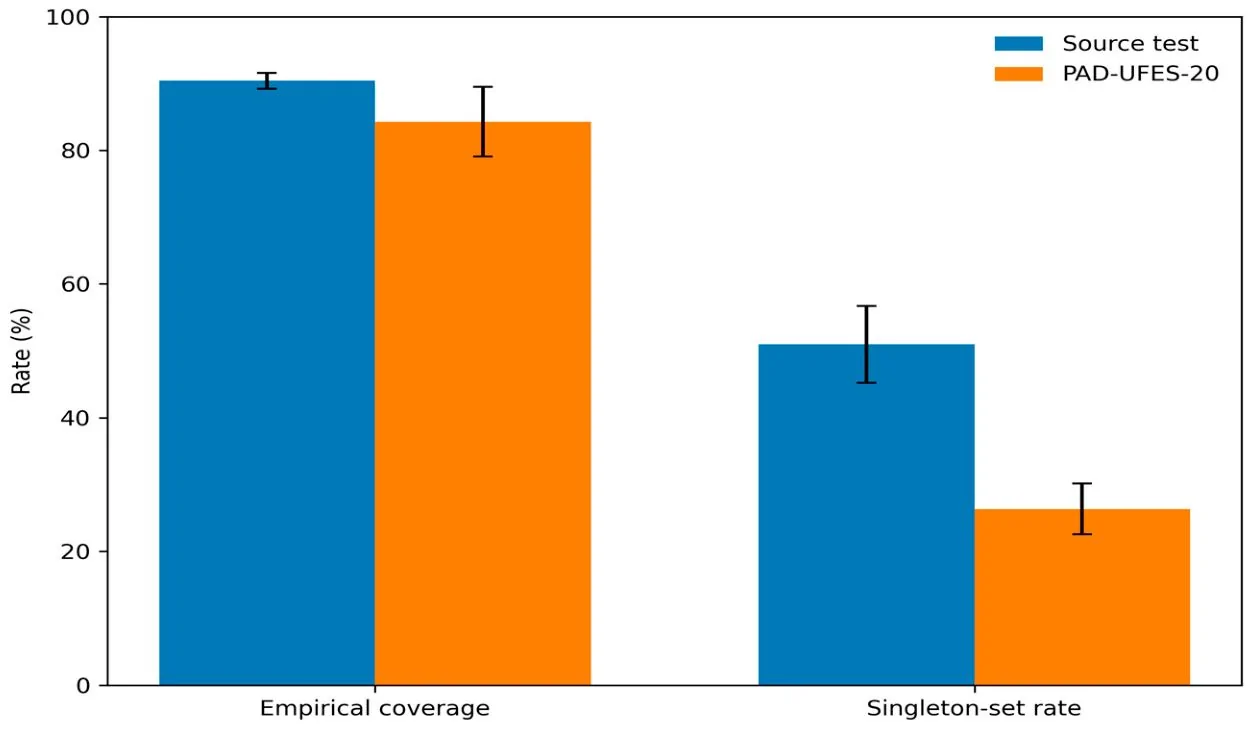
Conformal coverage and singleton prediction-set rates across the source and external domains.

PAD-UFES-20 showed lower empirical coverage, larger prediction sets, and a lower singleton-set rate than the source test sets as shown in Figure 8. The source-fitted selective policy also retained fewer external cases. These findings indicate reduced conformal efficiency and limited transfer of the source-derived selective policy. However, non-retained cases comprise both specialist-review and reliability-based deferral states and should not be interpreted as deferral alone.

**Figure 9:**
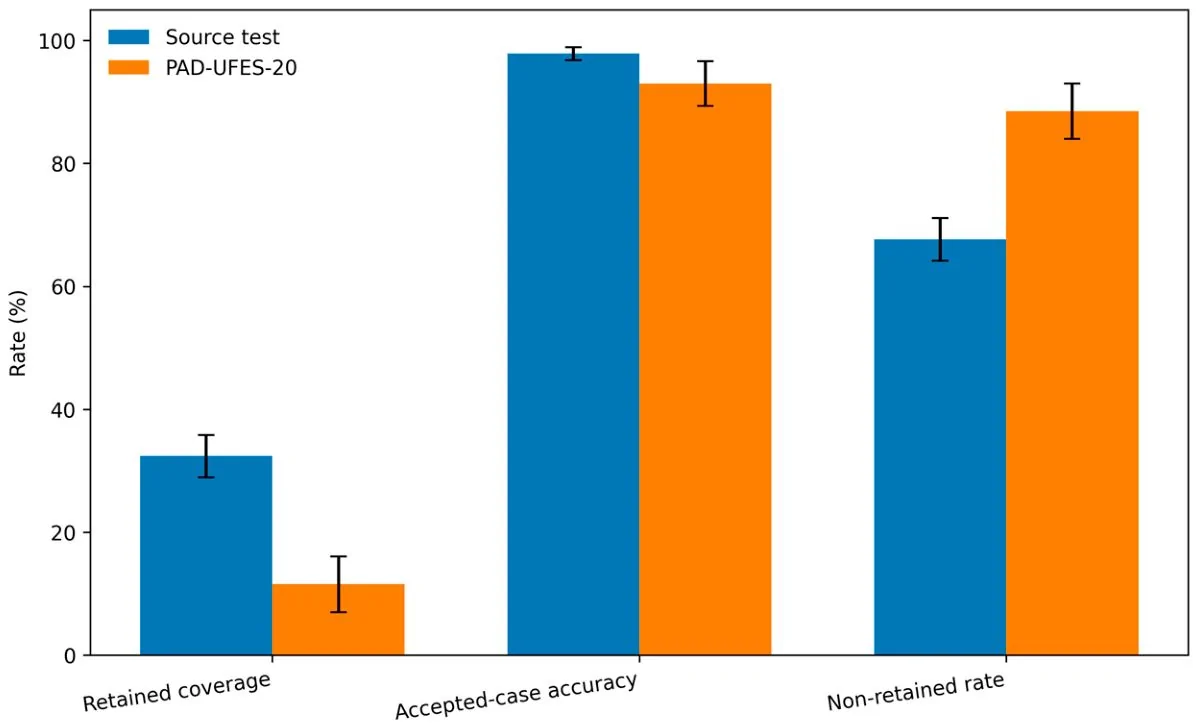
Retained coverage, accepted-case accuracy, and non-retained rate in the source-domain and PAD-UFES-20 test sets.

Figure 9 summarizes retained coverage, accepted-case accuracy, and non-retained rate across the source-domain and PAD-UFES-20 test sets. Accepted-case accuracy remained high in both domains, but retained coverage decreased from 32.37 ± 3.45% internally to 11.50 ± 4.53% externally. The corresponding non-retained rate increased from 67.63 ± 3.45% to 88.50 ± 4.53%. Because non-retained cases include both specialist-review and reliability-based deferral states, this quantity should not be interpreted as the deferral rate alone.

#### Selective-Baseline and Threshold-Sensitivity Analysis

To determine whether the composite gate provided information beyond simpler rejection strategies, confidence-only, entropy-only, and conformal-set-only baselines were evaluated using the same source-only fitting principle. Confidence and entropy thresholds were fitted on the source reliability-threshold partition at approximately the same prespecified retained-coverage target; conformal-only rejection retained singleton prediction sets. No PAD-UFES-20 observation was used to fit any baseline threshold.

In Table 9 Confidence-only and entropy-only rejection achieved accepted-case accuracy comparable to the composite score internally. Under PAD-UFES-20 transfer, however, the composite source-fitted score retained a larger fraction of cases than the confidence-only and entropy-only strategies while maintaining higher accepted-case accuracy and lower selective risk. Conformal-set-only rejection retained more external cases but at substantially higher selective risk. The composite policy is therefore not interpreted as uniformly superior at every source-domain operating point. Rather, it provided a more favorable external coverage–risk trade-off in this evaluation.

**Table 9:** Comparison of source-fitted selective-classification baselines.

Method	Domain	Retained (%)	Accepted Accuracy (%)	Selective Risk (%)
Composite	Source	32.37 ± 3.45	97.86 ± 1.04	2.14 ± 1.04
Confidence-only	Source	35.07 ± 1.32	98.08 ± 0.42	1.92 ± 0.42
Entropy-only	Source	35.18 ± 1.15	97.93 ± 0.31	2.07 ± 0.31
Conformal-only	Source	50.96 ± 5.73	89.89 ± 2.61	10.11 ± 2.61
Composite	PAD	11.50 ± 4.53	92.97 ± 3.64	7.03 ± 3.64
Confidence-only	PAD	7.43 ± 1.43	85.69 ± 4.35	14.31 ± 4.35
Entropy-only	PAD	7.25 ± 1.28	86.88 ± 5.23	13.12 ± 5.23
Conformal-only	PAD	26.35 ± 3.81	65.22 ± 6.63	34.78 ± 6.63

To assess the external behavior dependent on a single conservative operating point, the source-fitted selective-classification procedure was additionally evaluated across prespecified target retained-coverage levels of 25%, 35%, 45%, 55%, and 65%; the acceptance threshold was re-estimated exclusively from the source reliability-threshold partition. PAD-UFES-20 remained completely excluded from threshold fitting and was used only for post hoc evaluation of the resulting source-derived operating points.

Table 10 showed increasing the source-domain target coverage produced the expected coverage–risk trade-off. PAD-UFES-20 retained coverage increased from 7.62% at the 25% operating point to 34.29% at the 65% operating point, whereas accepted-case accuracy decreased from 93.76% to 87.33% and selective risk increased accordingly. This finding indicates that the externally low retained coverage at the primary 35% operating point was not attributable solely to a single excessively conservative threshold. Less conservative source-derived thresholds retained more external cases, but at progressively higher selective risk. Importantly, PAD-UFES-20 was used only for post hoc characterization and did not influence threshold fitting.

**Table 10:** Threshold-sensitivity analysis of the source-fitted selective-classification policy. Sensitivity of reliability-based deferral to the source-derived review threshold.

Target Source Coverage	Source Retained (%)	Source Accepted Accuracy (%)	Source Selective Risk (%)	PAD Retained (%)	PAD Accepted Accuracy (%)	PAD Selective Risk (%)
25%	25.89	98.16	1.84	7.62	93.76	6.24
35% (primary)	32.37	97.86	2.14	11.50	92.97	7.03
45%	44.83	96.93	3.07	19.71	91.47	8.53
55%	55.42	96.00	4.00	28.87	89.04	10.96
65%	60.92	95.21	4.79	34.29	87.33	12.67

The review threshold was varied only on the source reliability-threshold partition. PAD-UFES-20 values were computed post hoc after each source-derived threshold had been fixed and were not used for threshold selection as shown in Table 11.

**Table 11:** Sensitivity of reliability-based deferral to the source-derived review threshold.

Review-Threshold Quantile	Source Deferral (%)	PAD-UFES-20 Deferral (%)
70th	29.72 ± 1.61	53.76 ± 6.25
80th (primary)	20.59 ± 2.01	41.41 ± 5.99
90th	10.65 ± 1.76	27.75 ± 4.34

### 4.6. Demographic Subgroup Reliability

Demographic subgroup analyses were treated as reliability audits rather than as evidence of demographic fairness or equity. Sex-based discrimination and calibration were evaluated where sex metadata were available. Age-stratified external analyses were reconstructed by linking the fixed PAD-UFES-20 predictions to the corresponding locked metadata by image identifier. Skin-tone analysis was not performed because usable skin-tone metadata were unavailable in the evaluated cohorts. Consequently, no conclusions regarding skin-tone fairness or equity are made.

Across the source test sets, female and male subgroups showed similar class-balanced discrimination, although overall accuracy differed. On PAD-UFES-20, the female subgroup showed a higher mean macro-F1 than the male subgroup. However, this difference did not remain statistically significant after correction for multiple testing. The subgroup findings are therefore interpreted descriptively and as sensitivity analyses rather than as evidence of demographic parity.

Age-stratified PAD-UFES-20 results showed significant heterogeneity. Participants younger than 40 years had the highest overall accuracy but substantially lower balanced accuracy and macro-F1. This apparent discrepancy was strongly related to subgroup class composition: 159 of the 209 images in this age stratum were NEV, whereas only 2 MEL images were present. The youngest subgroup therefore cannot be adequately characterized by overall accuracy alone. Importantly, in the corrected analysis, this subgroup did not show the highest calibration error; its mean ECE was lower than that of the 40–59-year group.

**Figure 10:**
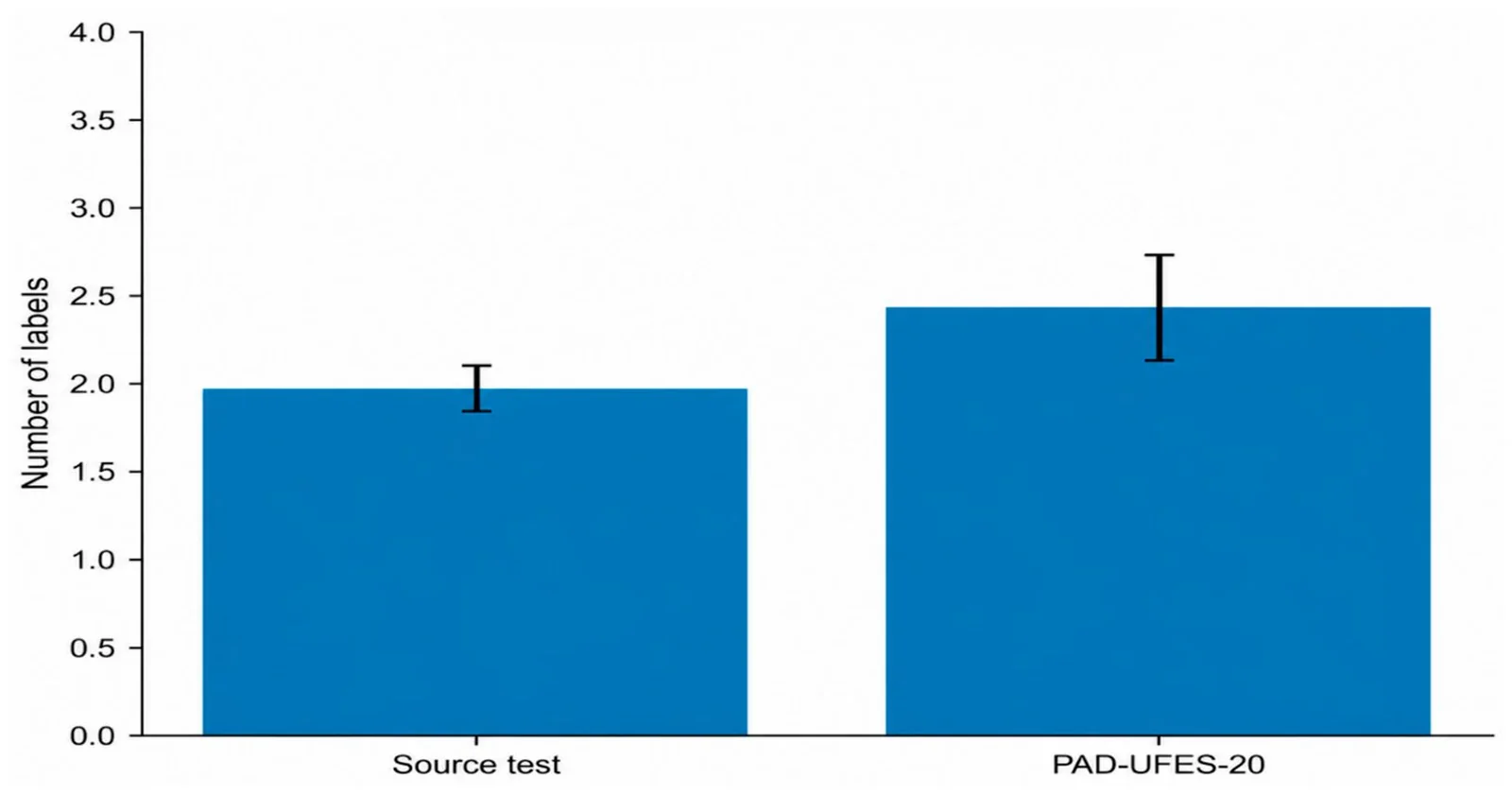
Demographic subgroup variation in discrimination and calibration across domains.

Figure 10 demonstrates marked heterogeneity across the PAD-UFES-20 age strata. The <40-year group showed the highest overall accuracy but the lowest balanced accuracy and macro-F1, illustrating the influence of its highly skewed class composition. In particular, NEV represented 159 of 209 images in this subgroup, whereas only 2 MEL images were available. Calibration did not follow the same pattern: ECE was 6.67% in the <40-year group, compared with 18.78% in the 40–59-year group and 9.86% in the ≥60-year group. These findings show that subgroup discrimination and calibration should be interpreted jointly and that subgroup composition can strongly affect apparently favorable aggregate accuracy.

Source-test subgroup sizes vary across seeds because the lesion/case-level test partitions were regenerated according to the predefined seed-specific partitions; source values therefore report the mean and range across the five seeds. PAD-UFES-20 subgroup membership was fixed across seeds. A total of 357 PAD-UFES-20 images had missing sex metadata and were excluded from the female–male comparison as shown in Table 12.

**Table 12:** Demographic subgroup composition.

Domain	Subgroup	Evaluated Images	Unique Cases	BCC	MEL	NEV	BK
Source	Female	508.2 mean [472–533]	363.0 [338–376]	3.9%	10.7%	76.0%	9.4%
Source	Male	608.6 mean [585–666]	430.6 [418–456]	5.8%	16.1%	66.1%	12.0%
PAD	Female	510	313	406 (79.6%)	28 (5.5%)	49 (9.6%)	27 (5.3%)
PAD	Male	509	290	439 (86.2%)	24 (4.7%)	26 (5.1%)	20 (3.9%)
PAD	Age < 40	209	147	40 (19.1%)	2 (1.0%)	159 (76.1%)	8 (3.8%)
PAD	Age 40–59	467	298	311 (66.6%)	27 (5.8%)	69 (14.8%)	60 (12.8%)
PAD	Age ≥ 60	700	433	494 (70.6%)	23 (3.3%)	16 (2.3%)	167 (23.9%)

Values are means with two-sided 95% confidence intervals across seeds 17, 29, 41, 53, and 71. These confidence intervals quantify variation across the five model runs and should not be interpreted as population-level patient confidence intervals as shown in Table 13.

**Table 13:** Demographic subgroup discrimination and calibration across five seeds.

Domain/Subgroup.	Accuracy %	Balanced Accuracy %	Macro-F1%	ECE %
Source Female	87.14 (85.31–88.97)	71.82 (67.65–75.99)	74.79 (70.47–79.11)	4.11 (3.15–5.07)
Source Male	83.22 (82.03–84.40)	72.51 (69.25–75.77)	74.00 (71.83–76.17)	3.68 (2.87–4.50)
PAD Female	62.86 (54.01–71.72)	60.18 (54.64–65.72)	45.73 (38.74–52.73)	12.69 (7.52–17.86)
PAD Male	59.21 (50.85–67.58)	56.95 (51.52–62.38)	37.22 (32.94–41.50)	14.47 (9.13–19.81)
PAD Age < 40	78.18 (76.10–80.27)	36.40 (31.39–41.40)	37.78 (33.08–42.49)	6.67 (5.29–8.05)
PAD Age 40–59	54.18 (45.00–63.35)	53.20 (47.77–58.63)	46.20 (39.61–52.79)	18.78 (11.17–26.38)
PAD Age ≥ 60	64.71 (58.45–70.98)	54.84 (52.50–57.18)	42.33 (40.10–44.57)	9.86 (6.33–13.40)

#### Statistical Sensitivity Analysis of Demographic Subgroups

Because subgroup estimates were available from only five matched training seeds, statistical inference was treated as sensitivity analysis rather than as confirmatory demographic inference. Female–male comparisons were evaluated using seed-matched paired tests, exact two-sided Wilcoxon signed-rank tests, and bootstrap confidence intervals, with Holm correction across the four prespecified subgroup metrics within each domain.

In the source test sets, female accuracy exceeded male accuracy by a mean of 3.93 percentage points. The paired t-test yielded *p* = 0.0019, and the Holm-adjusted *p*-value was 0.0074. However, the exact Wilcoxon test yielded *p* = 0.0625. Balanced accuracy, macro-F1, and ECE did not show consistent sex-related differences.

On PAD-UFES-20, female macro-F1 exceeded male macro-F1 by 8.51 percentage points. The unadjusted paired t-test yielded *p* = 0.0153, but this difference did not remain significant after Holm correction (adjusted *p* = 0.0613), and the exact Wilcoxon test also yielded *p* = 0.0625. We therefore describe this as an observed external subgroup difference rather than evidence of a statistically established sex disparity.

Across the three PAD age strata, Friedman tests indicated overall heterogeneity in accuracy (*p* = 0.0067; Holm-adjusted *p* = 0.0270), balanced accuracy (*p* = 0.0224; adjusted *p* = 0.0450), macro-F1 (*p* = 0.0408; adjusted *p* = 0.0450), and ECE (*p* = 0.0150; adjusted *p* = 0.0450). However, no individual pairwise age-group comparison was considered definitive because the exact Wilcoxon tests were limited by only five paired runs. Accordingly, the age findings are interpreted as evidence of overall subgroup heterogeneity rather than as proof that any specific age group is systematically disadvantaged.

### 4.7. Independent OOD and Explanation Stability Audit

Using the fixed Seed-17 checkpoint of the primary diagnostic architecture, OOD discrimination on unsupported DDI diagnoses was limited. One-minus maximum softmax probability achieved AUROC/AUPRC/FPR95 of 0.502/0.251/0.972, predictive entropy 0.515/0.283/0.969, and energy 0.599/0.385/0.969. Energy showed the highest separation among the three scores, but its absolute OOD performance remained modest.

Explanation stability was evaluated descriptively on 16 prespecified images per domain. Combined ESS was 0.751 ± 0.183 for the source test subset, 0.744 ± 0.143 for PAD-UFES-20, and 0.708 ± 0.209 for diagnosis-compatible DDI. These values quantify perturbation stability only and should not be interpreted as evidence of diagnostic correctness, causal validity, or lesion-localization accuracy.

Table 14 demonstrates the OOD audit compared the fixed-source test predictions for Seed-17 with unsupported DDI diagnoses using the core diagnostic architecture. Higher AUROC and AUPRC values indicate better OOD separation, while a lower FPR95 is preferred. The DDI dataset was not used in model fitting, calibration, matching, or selective threshold optimization.

**Table 14:** Independent post-development OOD detection on unsupported DDI diagnoses.

OOD Measure.	AUROC	AUPRC	FPR95
1 − Maximum softmax probability	0.502	0.251	0.972
Predictive entropy	0.515	0.283	0.969
Energy score	0.599	0.385	0.969

**Figure 11:**
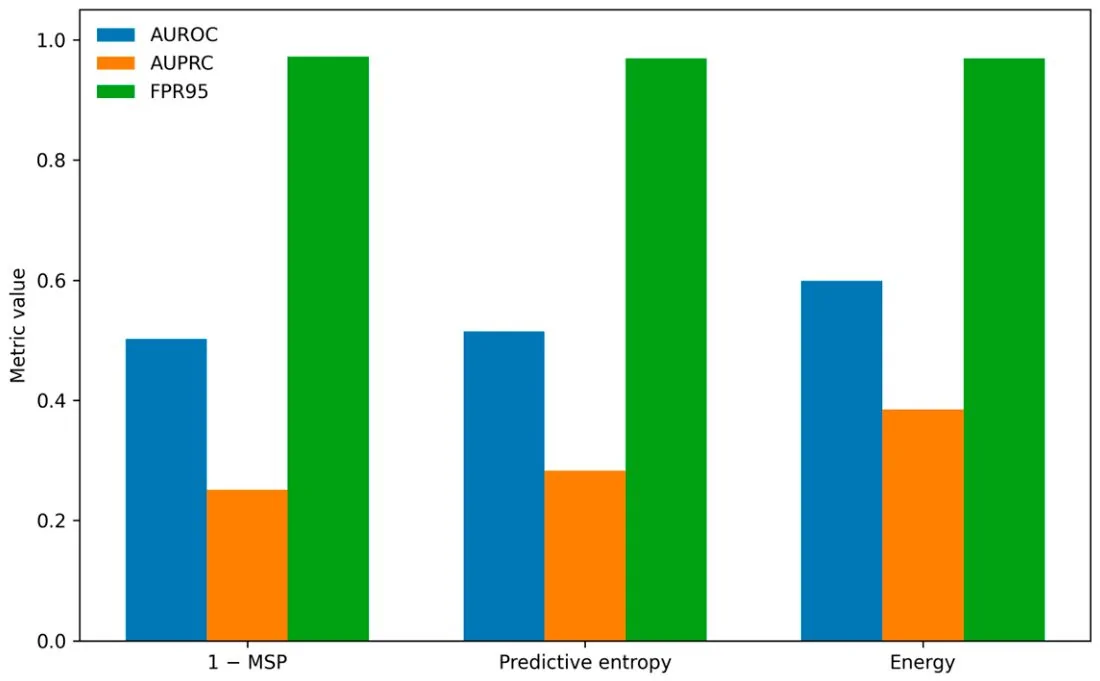
Independent post-development OOD assessment of unsupported DDI diagnoses using the fixed Seed-17 checkpoint of the primary diagnostic architecture. The internal source-test set served as the in-distribution reference, while DDI diagnoses outside the harmonized four-class label space formed the OOD challenge. Energy provided the highest discrimination among the three evaluated scores, although overall OOD separation remained limited.

Using the fixed checkpoint Seed-17 in the core diagnostic architecture, the distinction between unsupported diagnoses for Figure 11. Independent post-development OOD assessment of unsupported DDI diagnoses using the fixed Seed-17 checkpoint of the primary diagnostic architecture. The internal source-test set served as the in-distribution reference, and DDI diagnoses outside the harmonized four-class label space formed the OOD challenge. The maximum softmax probability measured one minus the maximum achieved AUROC/AUPRC/FPR95 values of 0.502/0.251/0.972, predictive entropy of 0.515/0.283/0.969, and energy of 0.599/0.385/0.969. Energy showed the highest separation between the three evaluated scores, but its absolute performance in distinguishing unsupported diagnoses remained modest.

The source-domain test set was used as in-distribution data, and unsupported DDI diagnoses were used as out-of-distribution samples. The dashed diagonal line indicates chance-level discrimination.

**Figure 12:**
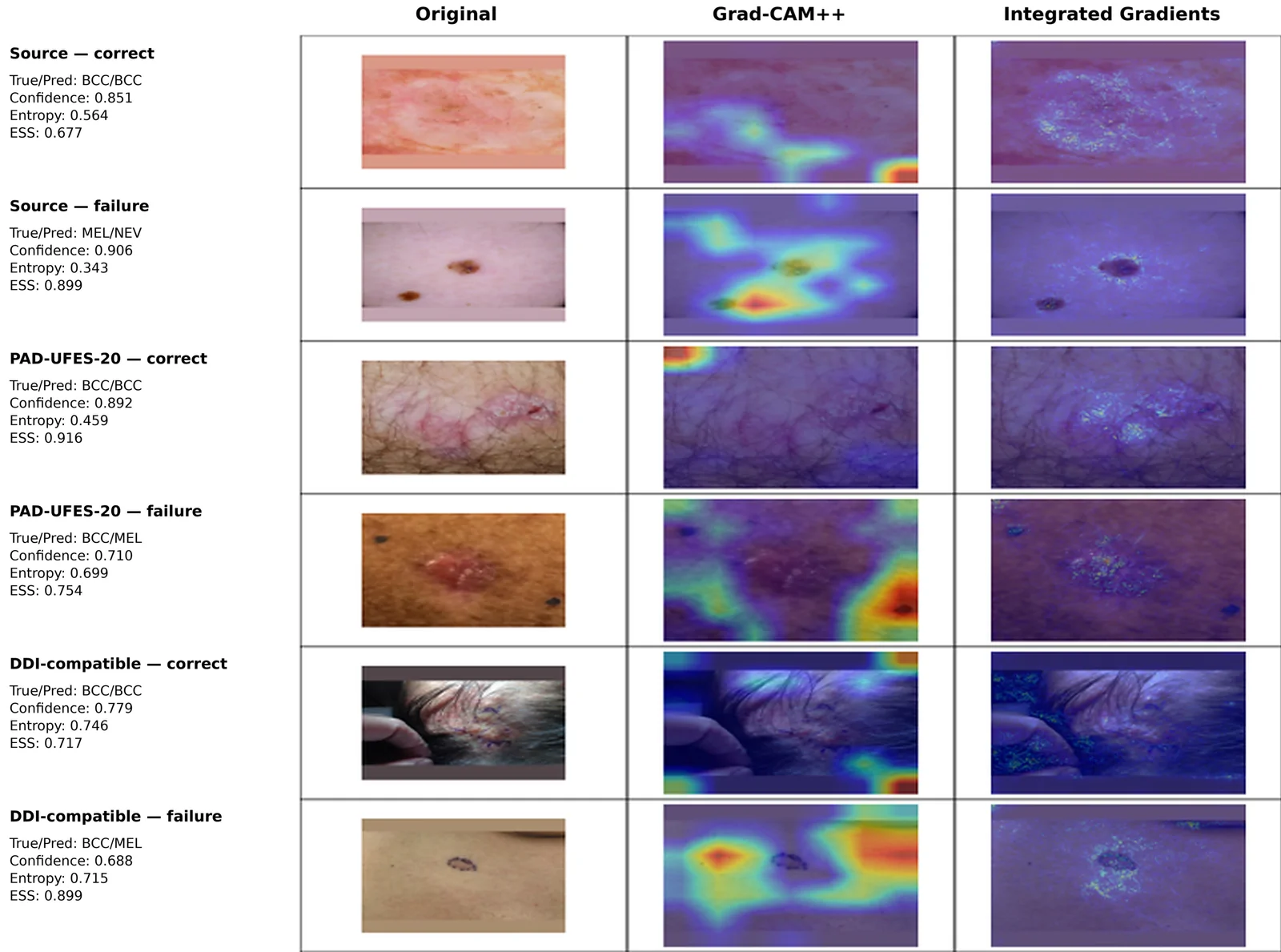
Cross-domain explainability audit of correct predictions and failure cases using the fixed Seed-17 primary-model checkpoint.

Table 15 summarizes values are mean ± standard deviation across the 16 audited images within each domain. ESS summarizes perturbation stability only and should not be interpreted as evidence of lesion localization, diagnostic correctness, causal validity, or clinical relevance. Explanation stability was evaluated descriptively on 16 prespecified images per domain. Combined ESS was 0.751 ± 0.183 for the source test subset, 0.744 ± 0.143 for PAD-UFES-20, and 0.708 ± 0.209 for diagnosis-compatible DDI images. These values quantify perturbation stability only and should not be interpreted as evidence of diagnostic correctness, causal validity, or lesion-localization accuracy. We present representative images from the source domain, PAD-UFES-20, and diagnosis-compatible DDI, along with attribution maps obtained using Grad-CAM++ and Integrated Gradients from the fixed Seed-17 checkpoint of the primary diagnostic architecture. To avoid interpreting explainability solely in terms of success, we present one correctly classified case and one misclassified case for each domain. For each case, we report confidence, predictive entropy, and the combined Explanation Stability Score (ESS). High ESS suggests that the attribution order is robust to perturbations but does not imply that the lesion is clinically relevant or that the attribution is causally valid. Figure 12 shows a balanced qualitative audit of explanation behavior across the source, unseen smartphone, and independent DDI domains. The figure is not a success-only visualization, as it includes both correctly classified and misclassified cases. Grad-CAM++ generally showed broader spatial activation patterns, while Integrated Gradients produced more localized attribution structures. Importantly, indicating that attribution stability alone does not guarantee diagnostic correctness. Thus, robustness was taken to mean that the explanation was stable, not that it provided direct evidence of clinical relevance or causal validity.

**Table 15:** Explanation of stability under diagnosis-preserving perturbations.

Domain	*n*	Grad-CAM++ Stability	Integrated Gradients Stability	Combined ESS
Source test	16	0.611 ± 0.380	0.891 ± 0.033	0.751 ± 0.183
PAD-UFES-20	16	0.666 ± 0.240	0.821 ± 0.090	0.744 ± 0.143
DDI-compatible subset	16	0.540 ± 0.387	0.876 ± 0.061	0.708 ± 0.209

For the source domain, the correct example (ISIC_0026118) had true/predicted label BCC/BCC, confidence 0.851, predictive entropy 0.564, and ESS 0.677; the failure case (ISIC_0031406) had MEL/NEV, confidence 0.906, entropy 0.343, and ESS 0.899. For PAD-UFES-20, the correct example (PAT_245_376_197) had BCC/BCC, confidence 0.892, entropy 0.459, and ESS 0.916; the failure case (PAT_736_1392_562) had BCC/MEL, confidence 0.710, entropy 0.699, and ESS 0.754. For diagnosis-compatible DDI, the correct example (000512) had BCC/BCC, confidence 0.779, entropy 0.746, and ESS 0.717; the failure case (000633) had BCC/MEL, confidence 0.688, entropy 0.715, and ESS 0.899. High ESS in both correct and incorrect cases demonstrates that explanation stability should not be interpreted as diagnostic correctness or causal validity. Qualitative inspection of the misclassified examples did not reveal a single recurrent non-pathological artifact that consistently explained the failures across domains. In several failure cases, Grad-CAM++ exhibited relatively broad activation extending into perilesional or surrounding image regions, whereas Integrated Gradients remained more spatially concentrated. However, formal artifact annotations for features such as hair, illumination glare, ruler marks, or background structures were not available in the audit subsets. We therefore do not attribute these errors to any specific artifact acquisition. Rather, the examples illustrate that a spatially stable attribution pattern can coexist with an incorrect diagnostic prediction and that perturbation stability alone does not establish clinically appropriate feature use.

**Figure 13:**
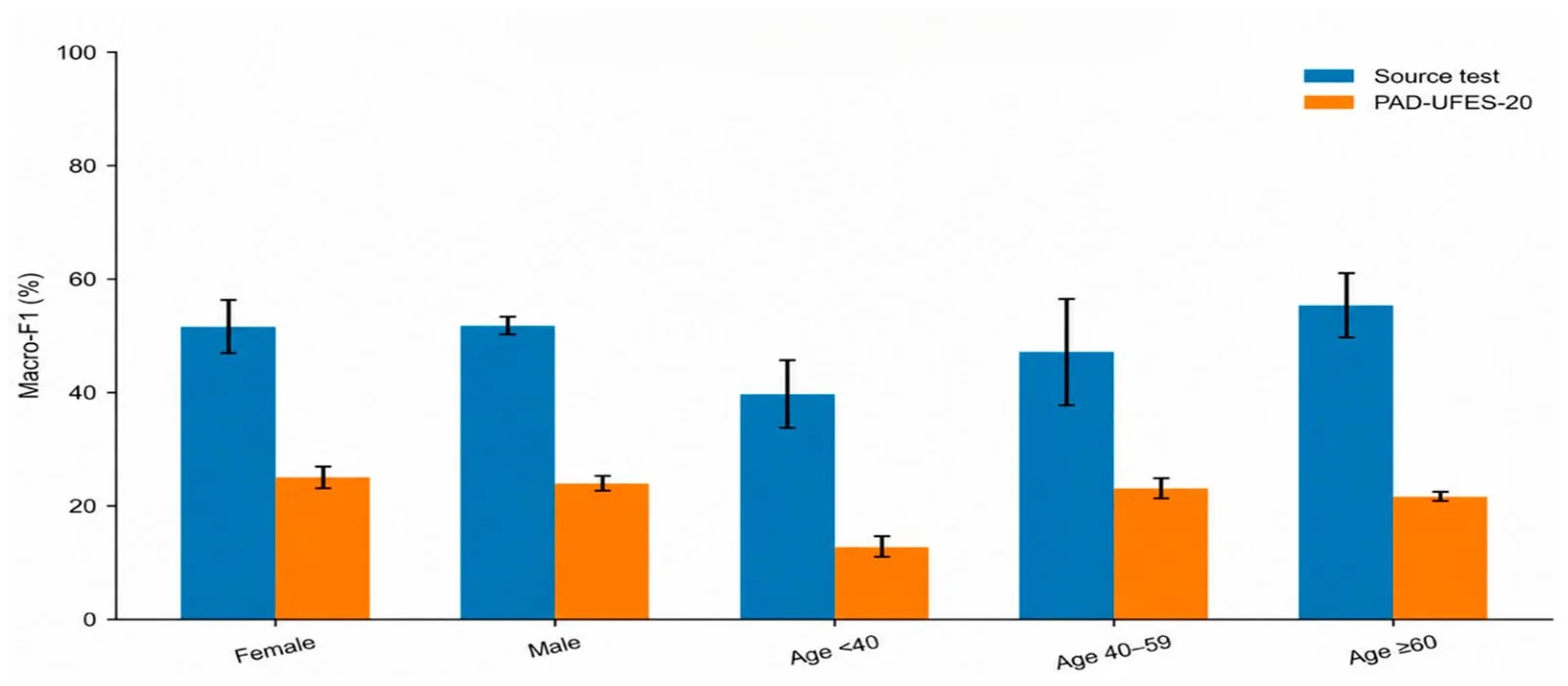
Cross-domain subgroup variation in macro-F1 score.

**Figure 14:**
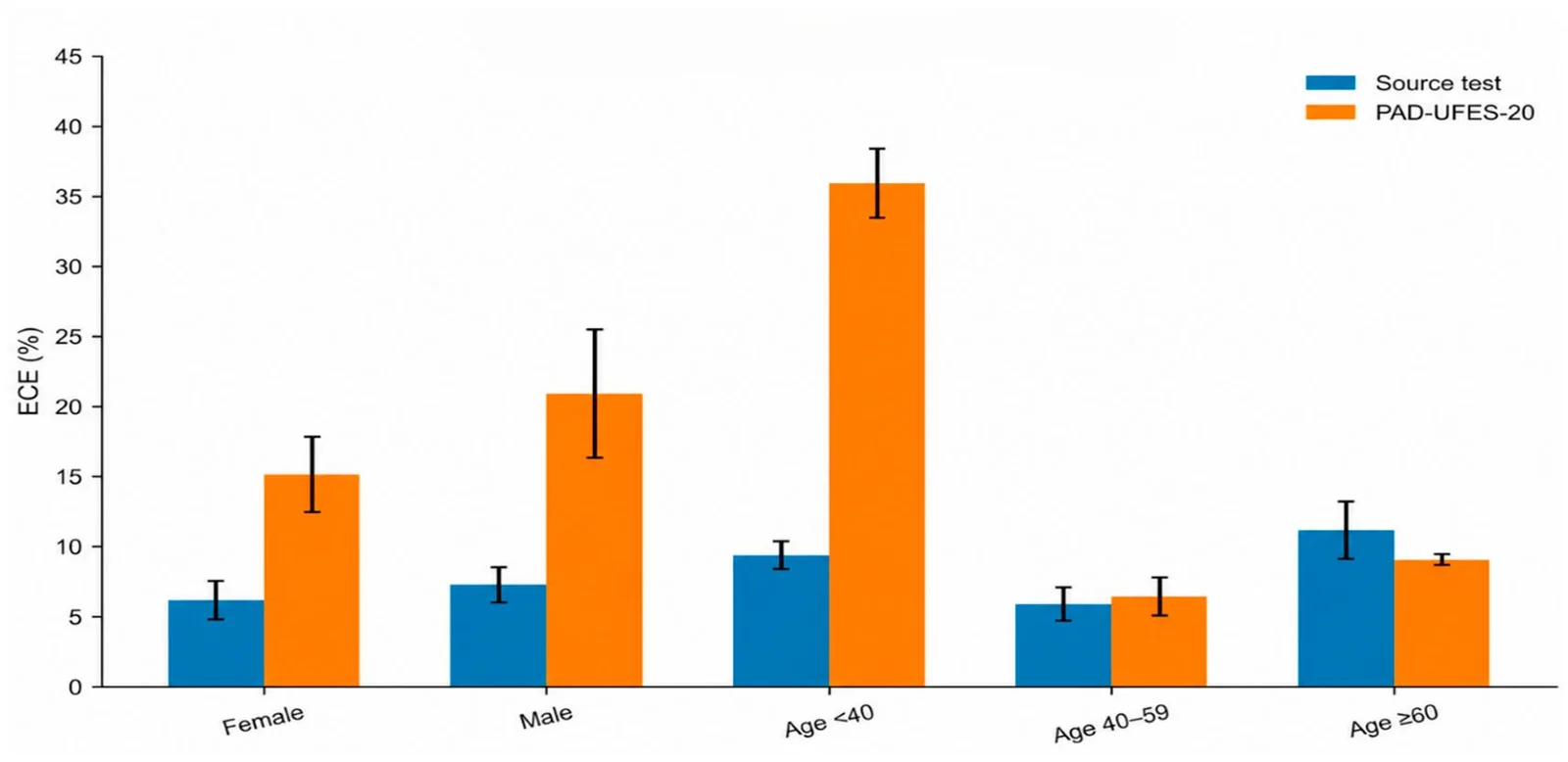
Cross-domain subgroup variation in expected calibration error.

High ESS values were observed in both correct and incorrect predictions as shown in Figure 13 and Figure 14. Therefore, attribution stability was interpreted as perturbation robustness rather than as evidence of diagnostic validity.

### 4.8. Conditional-Alignment Sensitivity Analysis

Conditional source alignment was examined as an exploratory single-seed sensitivity analysis. On PAD-UFES-20, alignment increased balanced accuracy from 24.84% to 31.22%, MCC from −0.013 to 0.096, and macro-AUROC from 0.569 to 0.589. However, external accuracy decreased from 53.42% to 30.96%, macro-F1 decreased from 22.99% to 21.15%, and ECE increased from 9.58% to 23.03%.

The alignment configuration redistributed classification errors and reduced majority-class dominance but did not consistently improve discrimination or calibration. One plausible explanation for the divergence between overall and balanced accuracy is the strong class imbalance in PAD-UFES-20: overall accuracy is heavily influenced by performance on the dominant BCC class, whereas balanced accuracy weights sensitivity equally across diagnostic classes. Diagnosis-conditional alignment may therefore have redistributed predictions away from the majority class and improved recall for some less prevalent classes while simultaneously increasing the absolute number of errors among majority-class samples. This would increase balanced accuracy despite reducing overall accuracy. The accompanying decreases in macro-F1 and the marked increase in ECE indicate that this redistribution did not constitute a uniformly favorable improvement. Because the analysis was performed with a single seed and was not used for model selection, this interpretation remains hypothesis-generating rather than causal or confirmatory see Table 16.

**Table 16:** Exploratory effect of conditional source alignment.

Domain	Configuration	Accuracy (%)	Balanced Accuracy (%)	Macro-F1 (%)	MCC	AUROC	ECE (%)
Source	Diagnostic model	70.86	54.81	51.81	0.440	0.874	6.81
Source	Alignment configuration	73.01	50.05	50.33	0.425	0.824	3.80
PAD	Diagnostic model	53.42	24.84	22.99	−0.013	0.569	9.58
PAD	Alignment configuration	30.96	31.22	21.15	0.096	0.589	23.03

### 4.9. Controlled Architectural Ablation

A controlled five-seed architectural ablation study was conducted to quantify the contribution of the main components of the proposed model under identical experimental conditions. All ablation variants used the same source-domain partitions, random seeds, optimization protocol, and external PAD-UFES-20 evaluation procedure as the full model. Four configurations were examined: no transformer, in which transformer contextualization was replaced by mean aggregation of the projected multi-scale visual features; no metadata, in which the clinical-metadata branch was removed; mean fusion, in which the learned gated-fusion mechanism was replaced by fixed mean fusion; and CNN-only, in which both contextual transformerization and metadata fusion were removed. This design enabled component-level comparison against the full model while keeping the remaining training and evaluation conditions unchanged.

Removing clinical metadata produced the largest component-specific reduction in external macro-F1, while the CNN-only configuration showed a comparable decline. Removing the transformer had a smaller external effect, whereas replacing learned gated fusion with mean fusion produced little change. These results indicate that the strongest incremental contribution in this evaluation came from the metadata pathway, whereas the advantage of learning gating over simple mean fusion was modest. The CNN-only variant additionally provides the simpler CNN-based comparison requested under identical five-seed experimental conditions as detailed in Table 17.

**Table 17:** Five-seed controlled architectural interpretation of the primary model.

Configuration	Source Macro-F1	PAD Macro-F1	Source BA	PAD BA	Source AUROC	PAD AUROC
Full	74.27 ± 1.36	54.92 ± 2.60	76.49 ± 1.67	61.88 ± 1.31	0.926 ± 0.005	0.880 ± 0.011
No Transformer	74.95 ± 0.69	53.35 ± 3.24	76.98 ± 0.89	61.13 ± 2.15	0.930 ± 0.010	0.874 ± 0.018
No Metadata	68.36 ± 2.66	51.60 ± 1.59	73.40 ± 2.08	59.27 ± 1.88	0.901 ± 0.006	0.848 ± 0.012
Mean Fusion	73.71 ± 2.21	54.79 ± 1.69	76.28 ± 2.01	61.29 ± 1.65	0.919 ± 0.006	0.878 ± 0.009
CNN-only	73.59 ± 1.19	51.36 ± 2.92	74.60 ± 1.40	57.88 ± 1.69	0.926 ± 0.007	0.859 ± 0.018

## 5. Discussion

The external evaluation should be interpreted as a compound cross-dataset transfer rather than as a controlled device-only experiment. PAD-UFES-20 differs from the source data in image-acquisition modality, diagnostic prevalence, and dataset composition. In particular, the dominant diagnosis changes from NEV in the source cohort to BCC in PAD-UFES-20. The observed reductions in macro-F1, balanced accuracy, and MCC therefore cannot be causally attributed to smartphone acquisition alone. The class-specific analysis nevertheless shows that transfer effects were heterogeneous across diagnoses, while the comparatively smaller decrease in macro-AUROC indicates that part of the class-ranking information remained preserved despite deterioration in the final multi-class decisions. Second, the reliability mechanisms were informative about the source distribution, but not operationally sufficient after cross-device transfer. The source-fitted conformal prediction achieved near-nominal coverage, and selective gating discovered a subset with substantially increased accepted-case accuracy. However, external conformal coverage dropped, singleton prediction sets were rare, and the source-fitted selective policy retained 32.37 ± 3.45% of source-test cases and 11.50 ± 4.53% of PAD-UFES-20 cases. Although accepted-case accuracy remained high, the reduction in external retained coverage indicates limited transfer of the source-derived admission policy. Threshold-sensitivity analysis further showed that less conservative source-fitted operating points increased external retained coverage at the cost of progressively higher selective risk.

The independent DDI audit provided complementary but non-interchangeable evidence. Energy scoring achieved the highest OOD AUROC among the evaluated scores, but its AUROC of 0.599 and high FPR95 indicate limited unsupported-diagnosis separation. Explanation-stability measurements were comparable across domains but remained descriptive; high ESS was observed in both correct and incorrect predictions and therefore does not establish diagnostic correctness or causal localization. However, the remaining FPR95 was too high to warrant using energy as the sole acceptance criterion. Moreover, Grad-CAM++ and Integrated Gradients showed high perturbation stability across the audited domains. However, even in some incorrect predictions, stable attribution patterns were observed. Hence, the stability of explanation should be understood as a measure of robustness to acquisition-preserving perturbations and not as a proof of lesion relevance, diagnostic correctness, or clinical causality. From a clinical standpoint, the results do not support unrestricted automated diagnosis or immediate deployment across imaging devices. Instead, they support a conservative decision-support role in which predictive output is withheld whenever the supporting reliability evidence is insufficient. The primary selective analysis jointly considered calibrated confidence, predictive entropy, and conformal ambiguity. The independent DDI audit used a single prespecified Seed-17 checkpoint of the primary diagnostic architecture rather than all five primary checkpoints. Explanation stability was evaluated descriptively on 16 images per domain. Independently verified lesion masks were unavailable. Therefore, lesion-localization validity could not be quantified. Table 18 puts the present findings into context by comparing the proposed reliability-oriented evaluation framework with representative skin-lesion AI studies focusing on classification performance, domain transfer, uncertainty, explainability, fairness, or selective decision support.

The comparison shows that benchmark-oriented models achieved higher internal classification accuracy under unrestricted use, while the proposed framework provided a broader quantitative assessment of predictive reliability. Its main contribution is the joint evaluation of source-only cross-device transfer, calibration, uncertainty-based error detection, conformal prediction, independent OOD recognition, explanation stability, subgroup reliability, and selective deferral. The comparative strength of the framework is thus in reliability-controlled decision support rather than in unrestricted benchmark accuracy alone.

### Limitations

This study has several limitations. The cross-device analysis was limited to four harmonized diagnostic categories and thus does not represent the broader dermatological label space. Second, source data remained largely imbalanced, and demographic analyses were limited by metadata completeness and subgroup sample sizes. Third, external validation was assessed retrospectively on a single smartphone-acquired dataset. Fourth, we have examined conditional source alignment as a single-seed sensitivity analysis, and this should not be taken as confirmatory evidence. Fifth, the independent DDI audit was conducted using a single prespecified Seed-17 checkpoint of the primary diagnostic architecture rather than all five primary checkpoints. OOD and explanation-stability measurements were reported as independent post-development audit evidence and were not inputs to the selective-classification gate. A major limitation of the XAI component is the absence of independently verified lesion masks across the audited cohorts. Consequently, the present analysis could quantify the perturbation stability of Grad-CAM++ and Integrated Gradients but could not quantitatively determine whether attribution was concentrated within clinically relevant lesion regions. This limitation is important because high explanation-stability scores were observed in both correct and incorrect predictions. Therefore, stability should not be interpreted as evidence of lesion localization, explanation fidelity, diagnostic correctness, or causal validity. Future validation should incorporate independently verified expert lesion annotations and quantitative lesion-relevance measures. Demographic subgroup auditing was limited to attributes supported by sufficiently complete metadata. Sex was available for only part of PAD-UFES-20, age was reconstructed retrospectively from the locked metadata, and usable skin-tone labels were unavailable in the evaluated datasets. Consequently, this study does not support conclusions regarding skin-tone reliability, demographic parity, or demographic equity.

## 6. Conclusions

We evaluated a multimedia classifier for skin lesions, categorizing them into four classes, using a strict external transfer protocol based solely on the source, utilizing the HAM10000 and Derm7pt datasets for development, and the PAD-UFES-20 dataset as an unseen external dataset derived from smartphone images. Across five prespecified random seeds, the overall F1 score in the source range was 74.27 ± 1.36%, and the overall AUROC value was 0.926 ± 0.005. While the overall F1 score in the PAD-UFES-20 dataset decreased to 54.92 ± 2.60%, the overall AUROC value remained relatively stable at 0.880 ± 0.011. Given the simultaneous change in acquisition conditions and the spread of diagnosis, these differences are interpreted as a compound external shift, rather than an isolated effect of the device.

The calibration specific to each category, conformal prediction, uncertainty-based error detection, and selective classification provided supplementary information on reliability. The selective policy suitable for the source retained fewer external cases while maintaining high accuracy in accepted cases, and the sensitivity analysis of the expected differential threshold confirmed the balance between coverage and risk. The independent DDI audit using a fixed checkpoint of the primary diagnostic architecture showed limited separation of unsupported diagnoses, with energy achieving an OOD AUROC of 0.599. Explanation-stability measurements remained descriptive and were not used to modify selective decisions. Demographic analyses identified heterogeneity among subgroups but did not demonstrate fairness or demographic equity. Controlled ablation showed that removing clinical metadata or limiting the configuration to only the convolutional neural network resulted in the largest decrease in the overall external F1 score. Overall, the results support explicit auditing of external reliability and selective decision-making under expert supervision, rather than assuming that internally reproducible discrimination guarantees reliable external performance.

## Figures and Tables

**Table 1:** Comparative analysis of prior studies on trustworthy and domain-generalizable multi-class skin-lesion AI.

Study	Data/Domain	Method or Study Design	Validation Setting	Key Finding/Contribution	Principal Limitation
[26] Daneshjou et al. (2022)	DDI; diverse clinical images	External fairness and robustness evaluation	Subgroup evaluation across skin tones and disease distributions	Revealed performance disparities on darker skin and uncommon diseases	No integrated calibration, explanation-stability, or triage analysis
[29] Singh et al. (2022)	Multi-class dermoscopy images lesion analysis	SkiNet with segmentation, uncertainty estimation, and XAI	Benchmark evaluation	Integrated uncertainty estimation and explainability within one framework	No cross-device fairness, class-wise calibration, or attribution-stability analysis
[32] Chamarthi et al. (2024)	Cross-dataset dermoscopy images classification	Unsupervised domain adaptation	Source-to-target adaptation using unlabeled target data	Reduced performance degradation caused by domain shift	Required access to the target domain and focused on a binary task
[34] Ieracitano et al. (2025)	Explainable skin-lesion classification	TIxAI attribution-relevance index	Lesion-region attribution evaluation	Quantified explanation relevance inside versus outside lesion regions	Did not assess attribution stability under device or acquisition changes
[35] Abdar et al. (2021)	Two skin-cancer image datasets	Bayesian uncertainty estimation and three-way decision	Two-dataset benchmark evaluation	Quantified uncertainty and supported rejection of ambiguous predictions	No multi-class cross-device fairness or explanation-reliability evaluation
[37] Murali and Mazumder (2026)	HAM10000; seven-class dermoscopy images classification	CNN–Transformer, dual attention, metadata fusion, and XAI	Within-dataset benchmark evaluation	Integrated local, global, and clinical features in an explainable multi-class model	No unseen smartphone-domain evaluation or integrated reliability analysis
[39] Zhu et al. (2026)	Skin-cancer images; demographic fairness	Lesion-guided attention and multi-objective optimization	Subgroup fairness evaluation	Reduced demographic disparity while preserving predictive performance	Restricted to a specific fairness attribute and task
[36] Chanda et al. (2024)	Melanoma–nevus assessment with dermatologists	Dermatologist-like visual and concept-based explanations	Human-reader evaluation	Improved clinician confidence and trust in AI-supported decisions	Limited to a controlled binary diagnostic setting
[40] Bhattacharyya et al. (2026)	Skin-lesion predictions across demographic groups	Conformal prediction and fairness assessment	Subgroup conformal evaluation	Quantified individual- and group-level uncertainty using prediction sets	No cross-device generalization or attribution-stability assessment
[41] Shafiq et al. (2026)	Multi-class clinical dermatology images	Feature fusion and explainable deep learning	Multi-dataset evaluation	Improved feature integration and model transparency	No strict unseen-domain generalization or subgroup calibration
[43] Somfai et al. (2023)	Dermoscopy images and clinical-image datasets	Model ensembles	Cross-dataset comparison	Demonstrated persistent dependence on the development dataset	Did not explicitly learn domain-invariant representations
[45] Tjiu and Lu (2025)	Clinical, dermoscopy images, and smartphone images	Systematic review and meta-analysis	Cross-setting evidence synthesis	Identified reduced performance in smartphone settings and across darker skin tones	Identified the problem without evaluating an integrated technical solution

**Table 18:** Comparison with representative skin-lesion AI frameworks.

Study	Dataset and Evaluation Setting	Main Reported Performance	Reliability Evidence	Main Limitation
Gessert et al. [22]	HAM10000; independent internal test set	MC-sensitivity: 75.7 ± 1.3%; MC-specificity: 96.0 ± 0.0%; F1-score: 82.6 ± 1.3%	Patch-based attention and diagnosis-guided class balancing	No external cross-device evaluation, probability calibration, OOD detection, fairness audit, or quantitative explanation-stability assessment
Gessert et al. [28]	ISIC 2019; cross-validation and official challenge test	Balanced accuracy: 74.2% in cross-validation and 63.4% on the official test	Multi-resolution ensemble with metadata	No integrated uncertainty, calibration, OOD, fairness, or selective prediction
Singh et al. [29]	ISIC 2018; internal benchmark	Accuracy: 88.46%; uncertainty-guided diagnostic accuracy: 73.65%	Bayesian uncertainty and multiple post hoc XAI methods	No unseen smartphone evaluation, conformal prediction, or subgroup analysis
Xin et al. [30]	HAM10000 and an independent dermoscopy images dataset	Accuracy: 94.3% and 94.1%, respectively	Transformer-based representation	No calibration, OOD, fairness, quantitative XAI stability, or selective triage
Chamarthi et al. [32]	Cross-dataset skin-lesion classification	Reduced domain-shift degradation using unsupervised domain adaptation	Target-assisted domain adaptation	Requires access to target-domain data before evaluation
Ieracitano et al. [34]	Explainable skin-lesion classification	Quantified lesion-related attribution using TIxAI	Quantitative explanation relevance	No cross-device explanation-stability, calibration, OOD, or selective-risk analysis
Murali and Mazumder [37]	HAM10000; internal seven-class evaluation	Accuracy: 94.8%; macro-F1: 91.9%; AUC: 0.957	Multimodal learning and post hoc XAI	No unseen smartphone-domain, calibration, OOD, or subgroup-reliability evaluation
Zhu et al. [39]	Skin-cancer images; subgroup evaluation	Improved demographic fairness while preserving predictive performance	Fairness-aware attention and multi-objective optimization	Restricted fairness setting; no unified conformal, OOD, or XAI-stability analysis
Bhattacharyya et al. [40]	Skin-lesion predictions across demographic groups	Quantified individual- and subgroup-level uncertainty	Conformal prediction and fairness evaluation	No strict unseen-device evaluation, OOD detection, or attribution-stability analysis
Somfai et al. [43]	Dermoscopy images and clinical-image datasets	Ensemble learning reduced, but did not eliminate, dataset dependence	Cross-dataset robustness analysis	No integrated calibration, fairness, OOD, or reliability-guided deferral
Proposed framework	HAM10000 + Derm7pt source development; unseen PAD-UFES-20 evaluation; independent DDI audit; five seeds	Source accuracy: 82.25 ± 1.76%; macro-F1: 74.27 ± 1.36%; macro-AUROC: 0.926 ± 0.005. On PAD-UFES-20, macro-F1 was 54.92 ± 2.60% and macro-AUROC was 0.880 ± 0.011. Accepted-case accuracy was 97.86 ± 1.04% at 32.37 ± 3.45% retained source coverage	Predictive-entropy error-detection AUROC: 0.821 ± 0.008; source conformal coverage: 90.39 ± 1.18%; independent DDI energy OOD AUROC: 0.599; source ESS: 0.751 ± 0.183; demographic subgroup reliability audit and five-seed architectural ablation	External full-coverage discriminations and selective coverage remained sensitive to the combined acquisition and diagnostic-prevalence shift represented by PAD-UFES-20

## Data Availability

The datasets analyzed in this study are available from their original public or research-access repositories. HAM10000 is available through Harvard Dataverse/ISIC (DOI: 10.7910/DVN/DBW86T); Derm7pt release_v0 is available through the official Simon Fraser University Derm7pt portal; PAD-UFES-20 Version 1 is available through Mendeley Data (DOI: 10.17632/zr7vgbcyr2.1); and DDI is available through the Stanford AIMI/DDI research portal (DOI: 10.71718/kqee-3z39). All resources were last verified for the present revision on 29 September 2026. No newly collected patient data are reported in this study.
